# High-Resolution Mapping in Two RIL Populations Refines Major “QTL Hotspot” Regions for Seed Size and Shape in Soybean (*Glycine max* L.)

**DOI:** 10.3390/ijms21031040

**Published:** 2020-02-04

**Authors:** Aiman Hina, Yongce Cao, Shiyu Song, Shuguang Li, Ripa Akter Sharmin, Mahmoud A. Elattar, Javaid Akhter Bhat, Tuanjie Zhao

**Affiliations:** 1 Ministry of Agriculture (MOA) Key Laboratory of Biology and Genetic Improvement of Soybean (General), State Key Laboratory for Crop Genetics and Germplasm Enhancement, Soybean Research Institute, National Center for Soybean Improvement, Nanjing Agricultural University, Nanjing 210095, China; aimanhina@yahoo.com (A.H.); songshiyu0706@126.com (S.S.); dawn0524@126.com (S.L.); ripa.sharmin@gmail.com (R.A.S.); mahmoud891987@gmail.com (M.A.E.); 2 Shaanxi Key Laboratory of Chinese Jujube; College of Life Science, Yan’an University, Yan’an 716000, China; caoyongce@yau.edu.cn

**Keywords:** Soybean, seed shape, seed size, QTL mapping, high-density genetic map, QTL hotspot, epistatic interactions, candidate genes

## Abstract

Seed size and shape are important traits determining yield and quality in soybean. However, the genetic mechanism and genes underlying these traits remain largely unexplored. In this regard, this study used two related recombinant inbred line (RIL) populations (ZY and K3N) evaluated in multiple environments to identify main and epistatic-effect quantitative trait loci (QTLs) for six seed size and shape traits in soybean. A total of 88 and 48 QTLs were detected through composite interval mapping (CIM) and mixed-model-based composite interval mapping (MCIM), respectively, and 15 QTLs were common among both methods; two of them were major (*R*^2^ > 10%) and novel QTLs (viz., *qSW-1-1_ZN_* and *qSLT-20-1_K3N_*). Additionally, 51 and 27 QTLs were identified for the first time through CIM and MCIM methods, respectively. Colocalization of QTLs occurred in four major QTL hotspots/clusters, viz., “QTL Hotspot A”, “QTL Hotspot B”, “QTL Hotspot C”, and “QTL Hotspot D” located on Chr06, Chr10, Chr13, and Chr20, respectively. Based on gene annotation, gene ontology (GO) enrichment, and RNA-Seq analysis, 23 genes within four “QTL Hotspots” were predicted as possible candidates, regulating soybean seed size and shape. Network analyses demonstrated that 15 QTLs showed significant additive x environment (AE) effects, and 16 pairs of QTLs showing epistatic effects were also detected. However, except three epistatic QTLs, viz., *qSL-13-3_ZY_*, *qSL-13-4_ZY_*_,_ and *qSW-13-4_ZY_*, all the remaining QTLs depicted no main effects. Hence, the present study is a detailed and comprehensive investigation uncovering the genetic basis of seed size and shape in soybeans. The use of a high-density map identified new genomic regions providing valuable information and could be the primary target for further fine mapping, candidate gene identification, and marker-assisted breeding (MAB).

## 1. Introduction

Soybean (*Glycine max* L.) is one of the most economically important crops, being a rich source of both edible oil and protein, and can fix atmospheric nitrogen through a symbiotic association with microorganisms in the soil, and are used as a model plant for legume research [1]. However, over the past five decades, a continuous decline in soybean production in China has been recorded [2]. Besides, annually, China imports more than 80% of soybeans and its products to meet its domestic demands; hence, there is an immediate need to increase the domestic production of soybean to make the country self-sufficient [2]. Yield-related traits are the key target of plant breeders to improve soybean yield/production. In this regard, traits related to seed size and shape are the crucial parameters determining seed-weight and yield in soybean [3,4]. In soybean, seed size traits such as length (SL); width (SW) and thickness (ST); and seed shape traits, viz., length-to-width (SLW), length-to-thickness (SLT), and width-to-thickness (SWT) ratios determine seed appearance, quality, and yield in soybeans [5]. Seed size is also a vital fitness trait in flowering plants and plays a crucial role in adaptation to a particular environment [6]. However, seed size and shape are complex quantitative traits governed by polygenes and highly influenced by the environment (E) and genotype × environment (G × E) interactions [7,8]. Specific soy-based food products made from soybean are also determined mainly by seed size and shape [9,10]. For example, for the production of fermented soybeans (natto) and sprouts, small-seeded cultivars are suitable, while for soymilk, green soybeans (edamame), boiled soybeans (nimame), and soybean curd (tofu), large-seeded varieties are used [11,12,13]. Additionally, these traits influence the germination ability and seedling vigor, and that, in turn, plays an essential role in determining the competitive strength of the seedlings for light, nutrient resources, and stress tolerance [14,15,16].

Quantitative trait loci (QTL) analysis has proved as a powerful technique to elucidate complex trait architecture. Over the past two decades, recent advances in marker technology and statistical methods have allowed the identification of many QTLs related to seed size and shape traits. The USDA Soybean Genome Database (SoyBase, http://www.soybase.org) presently document more than 400 QTLs for seed size and shape, and the majority of them are not confirmed (http://www.soybase.org). The previous studies used mostly low-resolution and low-density molecular markers such as simple sequence repeats (SSRs) that often result in larger confidence intervals and make the use of these QTLs less effective in crop improvement [3,5,17,18]. For example, Mian et al. [19] reported 16 QTLs for seed size and shape on 12 different chromosomes of soybean. Hoeck et al. [20] identified 27 QTLs associated with seed size distributed on 16 soybean chromosomes, and Li et al. [21] detected three QTLs for SL on Chr07, Chr13, and Chr16. Lü et al. [18] identified 19 main-effect QTLs (M-QTLs) and three epistatic-effect QTLs (E-QTLs) for SL on eight chromosomes. Xie et al. [22] finely mapped QTLs for soybean seed size traits on Chr06 in the recombinant inbred line (RIL) population derived from a cross between Lishuizhongzihuang and Nannong493-1. Likewise, Che et al. [17] identified 16 QTLs for seed shape, distributed on seven linkage groups in soybeans by using the RIL population. Hu et al. [7] mapped 10 QTLs for seed shape on six chromosomes in soybeans. However, only a few yield-related stable QTLs have been identified in different genetic backgrounds and environments [23]. Hence, it is vital to identify and validate QTLs in multiple backgrounds and environments for their potential use in marker-assisted breeding (MAB). Lastly, the earlier studies mostly focused on the identification of main-effect QTLs for seed size/shape in soybean; however, minimal efforts have been made to understand complex genetic interaction effects, such as epistasis and environment effects [24,25,26].

The inheritance of quantitative traits varies from simple to complex; however, the phenotypic variation of most quantitative traits is complex, governed by many factors [27]. In addition to main-effect QTLs, phenotypic variation (PV) of complex traits is also governed by QTL by QTL (epistatic) and QTL by environment (QTL × E) interactions, which contribute significantly to complex trait variations [28]. By considering these QTL interactions in the QTL mapping model of complex traits will lead to increased precision of QTL mapping [29]. Therefore, these factors cannot be considered only as the main obstacles to dissect the genetic architecture of complex traits, but they also affect the accuracy of breeding value estimation, and thus, hinder the efficiency of breeding programs. Hence, it is imperative to consider these factors while dissecting the genetic basis of complex traits and their uses in improving plant performance. In recent years, epistatic and QTL × E interaction effects are under consideration in several crop species, including soybeans, for QTL mapping [30]. Therefore, extensive efforts are required to study such QTL interaction effects for their effective exploitation in soybean breeding.

Development of high-density genetic maps, and their use in the detection of QTLs/genes, have allowed a detailed and broader understanding of the genetic basis underlying complex quantitative traits. Furthermore, the analysis of genes has partitioned the related traits into individual Mendelian factors [31]. Nevertheless, limited reports are targeting the mapping of QTLs related to seed size and shape based on the high-density map in different genetic backgrounds. Besides, to mine candidate genes for seed size and shape in soybeans, negligible efforts were made. By keeping the above in view, the present study has used a high-density linkage map of two RIL populations, viz., ZY and K3N, evaluated in multiple environments to identify main and epistatic-effect QTLs, as well as their interactions with the environment, to mine candidate genes for seed size and shape in soybeans. These results will be helpful in MAB for developing soybean varieties with improved yield and quality, as well as to clone underlying genes for seed size and shape in soybean.

## 2. Results

### 2.1. Evaluation of Phenotypic Variation for RIL Populations

Mean, range (minimum and maximum value), standard deviation, skewness, kurtosis, heritability (*h*^2^), and coefficient of variation (CV%) associated with six soybean seed shape and size traits of two RIL populations (ZY and K3N), along with their parents, evaluated across three different environments, viz., 2012FY, 2012JP, and 2017JP, are presented in Table S1.

**Figure 1:**
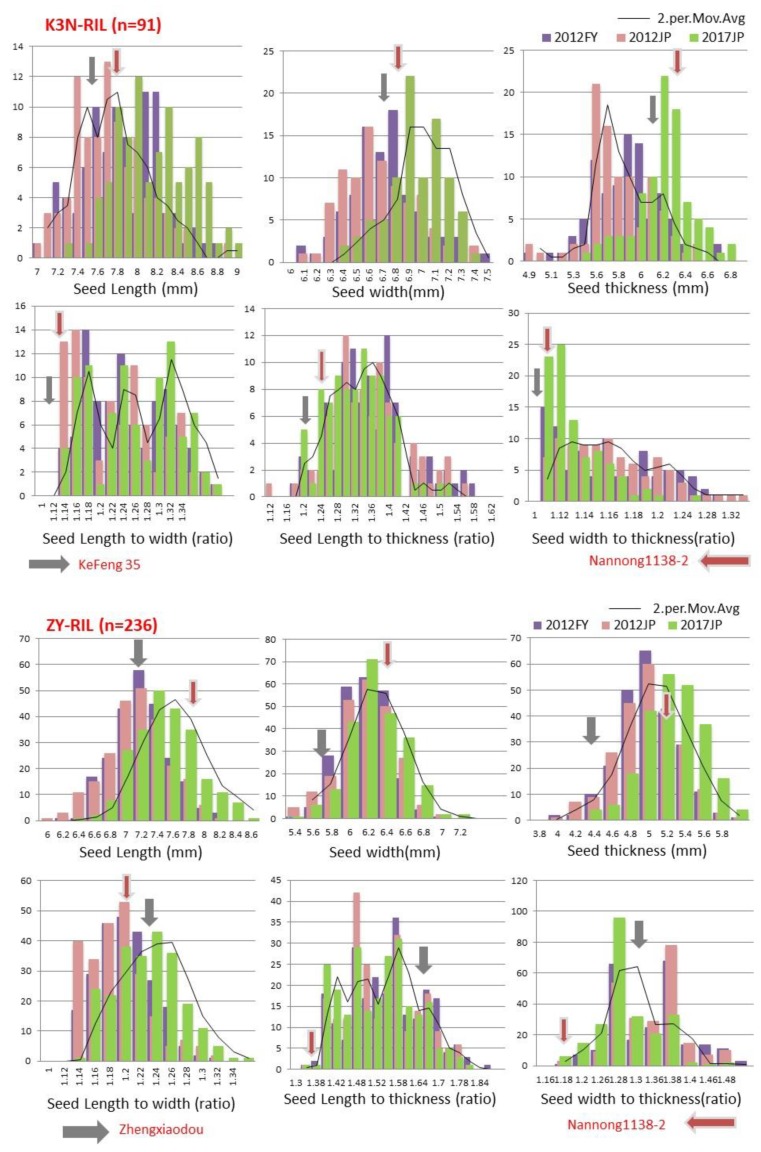
Frequency distribution of seed length (SL), seed width (SW), seed thickness (ST), seed length-to-width (SLW), seed length-to-thickness (SLT), and seed width-to-thickness (SWT) in ZY and K3N recombinant inbred line (RIL) populations across three different environments *(*2012FY, 2012JP, and 2017JP). Trend lines show the moving average. Arrows represent mean value of corresponding parent. Horizontal and vertical axis represent trait value and number of genotypes, respectively.

The difference in average phenotypic values between the contrasting parents of both RIL populations for all six traits was evident and consistent across all three individual environments (Table S1). The trait value of several RILs exceeded their parents for all studied traits in both directions, suggesting transgressive segregation in both RIL populations (Figure 1). All six traits related to seed size and shape showed different levels of distribution in both RIL populations (ZY and K3N), with mostly skewness and kurtosis <1, and the majority have CV >3%, which is typical for quantitative traits, indicating the suitability of these populations for QTL mapping (Figure 1 and Table S1a,b).

Combined ANOVA results revealed that variations among the RILs of both populations were highly significant (*p* < 0.0001 or *p* < 0.05) for all six traits (Table S2a,b). The environmental differences and G × E interaction effects were also highly significant for all the studied traits, except SLW, SLT, and SWT in the case of the K3N population (Table S2a,b). Heritability in a broad sense (*h*^2^) for both RIL populations in individual, as well as combined, environments was above 60%, indicating high heritability for all studied traits (Table S2a,b). The correlation coefficient (*r*^2^) among the six traits related to seed size and shape for both RIL populations are presented in Table S3. Correlation analysis has shown a significant positive correlation between any two seed shape size traits, and a significant negative correlation exists between seed shape and seed size traits (Table S3).

### 2.2. QTL Mapping of Seed Size by CIM

**Figure 2:**
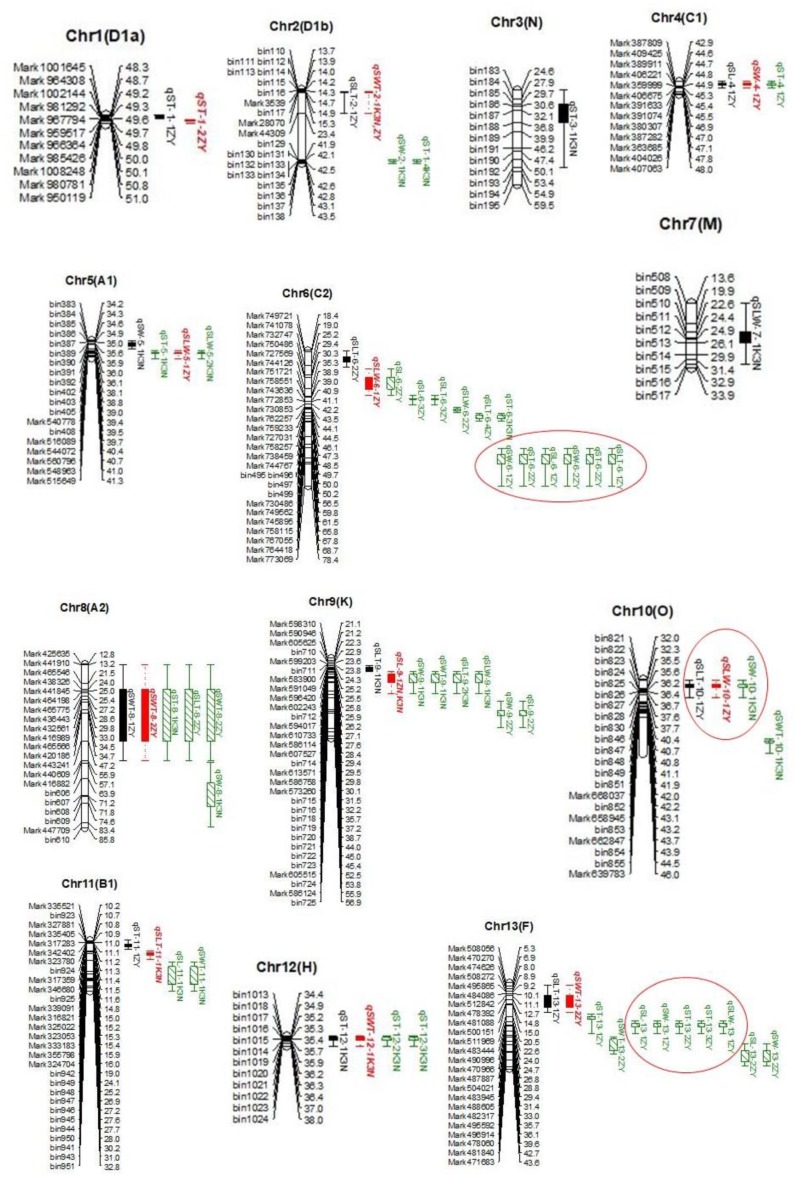

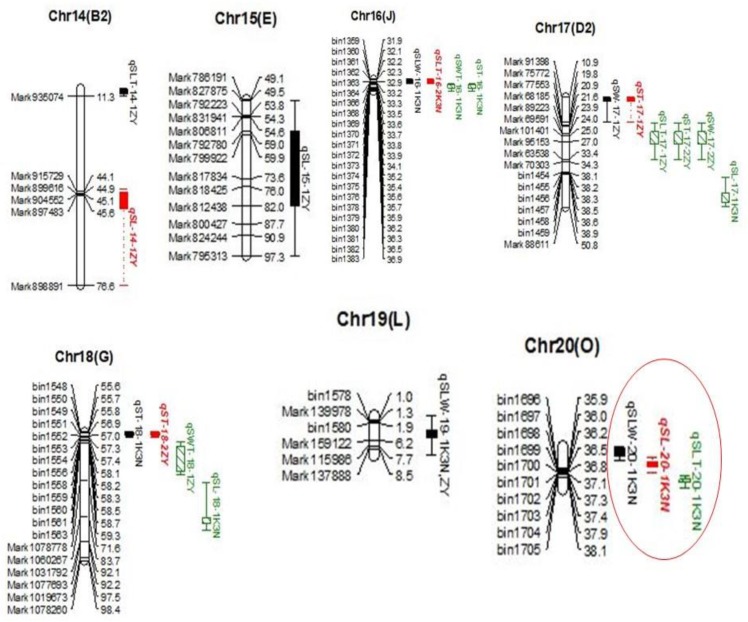
Location of quantitative trait loci (QTLs) on the genetic linkage map of the ZY and K3N RIL populations. Distances among markers are indicated using the physical location to the right of the linkage groups; names of markers are shown on the left. Only those SNP/SLAF markers are shown that were in and around the QTL regions. The red circles indicate the four QTL hotspots/clusters. Colored bars represent different QTLs.

The high-density genetic maps of ZY and K3N populations were used to perform a linkage analysis for the identification of QTLs associated with SL, SW, and ST in soybeans. In total, we identified 50 main-effect QTLs associated with three seed size traits, viz., SL, SW, and ST, explaining the phenotypic variation (PV/*R*^2^) of 4.46–22.64%, mapped on 18 soybean chromosomes in both ZY and K3N populations across three environments, viz., 2012FY, 2012JP, and 2017JP (Table 1 and Figure 2). For seed length (SL), 14 main-effect QTLs were detected on ten different chromosomes (Table 1). Among them, *qSL-9-1_ZY_*, *_K3N_* was stable and had significant QTL with an average *R*^2^ = 10.01% and are consistently found in two individual environments (2012FY and 2017JP), as well as in both RIL populations (ZY and K3N) (Table 1). Additionally, *qSL-13-1_ZY_*, expressing a PV of 8.26%, was detected in two different environments (2017JP and 2012JP) in the ZY population (Table 1). Moreover, one minor stable QTL, *qSL-4-1_ZY_*, expressing an average PV of 6%, was consistently identified in all three studied environments, viz., 2012FY, 2012JP, and 2017JP (Table 1). Four major QTLs, viz., *qSL-11-1_K3N_*, *qSL-17-1_K3N_*, *qSL-18-1_K3N_*, and *qSL-20-1_K3N_*, with *R*^2^ > 10%, were environmental-sensitive and identified in only one environment in the K3N population (Table 1). The remaining seven minor QTLs (*R*^2^ < 10%), viz., *qSL-6-1_ZY_*, *qSL-6-2_ZY_*, *qSL-6-3_ZY_*, *qSL-9-2_ZY_*, *qSL-13-2_ZY_*, *qSL-14-1_ZY_*, and *qSL-15-1_ZY_*, were also identified in a single environment in the ZY population (Table 1).

In both ZY and K3N populations, a total of 14 main-effect QTLs associated with SW were identified, distributed on ten different chromosomes/LG (Table 1). Among them, *qSW-13-1_ZY_* was detected in two individual environments, viz., 2012JP and 2017JP, in ZY population and expressed an average of 7.45% of PV (Table 1). However, nine major QTLs, viz., *qSW-2-1_K3N_*, *qSW-5-1_K3N_*, *qSW-6-1_ZY_*, *qSW-6-2_ZY_*, *qSW-8-1_K3N_*, *qSW-9-1_K3N_*, *qSW-10-1_K3N_*, *qSW-17-1_ZY_*, and *qSW-17-2_ZY_*_,_ with *R*^2^ > 10%, were identified only in one environment and expressed PV that varies from 10.33–17.32% in both RIL populations (Table 1). Four minor QTLs, viz., *qSW-1-1_ZY_*, *qSW-4-1_ZY_*, *qSW-9-2_ZY_*, and *qSW-13-2_ZY_*, were also detected as environment-sensitive and expressing a PV of 5.19–8.63% (Table 1).

For ST, we identified 22 main-effect QTLs in both RIL populations across three environments, distributed on 13 LG (Table 1). One stable major (*qST-6-2_ZY_*) and minor (*qST-13-3_ZY_*) QTLs were consistently detected in two individual environments in the ZY population with an average *R*^2^ of 11.32% and 6.36%, respectively (Table 1). Moreover, ten major QTLs: *qST-2-1_K3N_*, *qST-3-1_K3N_*, *qST-5-1_K3N_*, *qST-6-3_K3N_*, *qST-8-1_K3N_*, *qST-12-1_K3N_*, *qST-12-3_K3N_*, *qST-13-2_ZY_*, *qST-16-1_K3N_*_,_ and *qST-18-1_K3N_* were identified in only one individual environment in the K3N population, with PV ranging from 10.00–16.67% (Table 1). Besides, ten minor QTLs, viz., *qST-1-1_ZY_*, *qST-1-2_ZY_*, *qST-4-1_ZY_*, *qST-6-1_ZY_*, *qST-11-1_ZY_*, *qST-12-2_K3N_*, *qST-13-1_ZY_*, *qST-17-1_ZY_*, *qST-17-2_ZY_*, and *qST-18-2_ZY_*_,_ expressing PV in the range of 4.46–9.68%, were environment-sensitive (Table 1).

Among 50 QTLs identified for all three seed size traits, 31 QTLs were novel identified for the first time, and the remaining 19 QTLs are reported earlier in the same physical genomic interval (Table 1). Moreover, 25 out of 50 QTLs were major, with *R*^2^ > 10%, and the remaining 25 were minor QTLs, with *R*^2^ < 10%. However, we detected several major QTLs in the K3N population (18), compared to the ZY. Notably, the most prominent QTL with the highest logarithm of odds (LOD) score (10.76) in a 23.31cM region was located on Chr06, named *qSW-6-2_ZY_*, expressing 14.45% of PV (Table 1). The majority of QTLs showed a positive additive effect with favorable alleles from parent Zhengxiaodou, except ten QTLs (*qSL-9-2_ZY_*, *qSL-14-1_ZY_*, *qSL-18-1_K3N_*, *qSL-20-1_K3N_*, *qSW-2-1_K3N_*, *qSW-8-1_K3N_*, *qSW-10-1_K3N_*, *qST-2-1_K3N_*, *qST-6-3_K3N_*, and *qST-8-1_K3N_*) that displayed negative additive effects with beneficial alleles from Nannong1138-2 (Table 1).

### 2.3. QTL Mapping of Seed Shape by CIM

In total, we identified 38 QTLs associated with three seed shape traits, viz., SLW, SLT, and SWT on 15 different chromosomes in both RIL populations (ZY and K3N) across all three individual environments (Table 2 and Figure 2). A single QTL expressed a PV that varies from 3.44% (*qSLT-16-1_K3N_*) to 26.84% (*qSLW-20-1_K3N_*) (Table 2). For SLW, we identified 11 QTLs located on nine different chromosomes (Table 2). A major and stable QTL, *qSLW-6-1_ZY_*, was detected consistently on Chr06 in all three individual environments (2012FY, 2012JP, and 2017JP) in the ZY population and expressed a PV of 16.03% (Table 2). Besides, another major stable QTL, *qSLW-20-1_K3N_*, was identified on Chr20 in two individual environments (2012 JP and 2017JP), expressing an average PV of 19.24% in the K3N population (Table 2). The *qSLW-19-1_K3N_*,*_ZY_* was identified in both RIL populations, as well as two individual environments (2012FY and 2017JP), with an average PV of 9.17% (Table 2). The remaining eight QTLs were environment-sensitive (identified in only one individual environment); out of them, three QTLs, viz., *qSLW-7-1*_K3N_, *qSLW-9-1*_K3N_, and *qSLW-16-1*_K3N_, were major, with *R*^2^ > 10% (Table 2).

In the case of SLT, we identified a total of 16 QTLs distributed on 11 different chromosomes in both RIL populations across three individual environments (Table 2). Among them, *qSLT-10-1_ZY_* and *qSLT-20-1*_K3N_ were significant and stable QTLs having *R*^2^ > 10%, as well as detected in three and two individual environments, respectively (Table 2). Additionally, four significant QTLs, viz., *qSLT-9-1_K3N_*, *qSLT-9-2_K3N_*, *qSLT-11-1_K3N_*, and *qSLT-13-1_ZY_*, expressing a PV of 10.29–12.62%, were detected only in one individual environment (Table 2). The remaining ten QTLs were minor, having *R*^2^ < 10% detected in only one individual environment (Table 2).

For SWT, a total of 11 QTLs on nine different chromosomes were mapped in both RIL populations (Table 2). Among these QTLs, *qSWT-*2*-1_K3N_*,*_ZY_* and *qSWT-8-1_ZY_* were the stable QTLs identified in three and two individual environments, respectively; additionally, *qSWT-2-1_K3N_*,*_ZY_* was identified in both RIL populations. Besides, four out of 11 QTLs, viz., *qSWT-9-1_K3N_*, *qSWT-10-1_K3N_*, *qSWT-11-1_K3N_*, and *qSWT-16-1_K3N_*, were major (*R*^2^ > 10%) but were environment-sensitive, detected only in K3N-RIL populations (Table 2). The remaining five minor QTLs, viz., *qSWT-8-2_ZY_*, *qSWT-12-1_K3N_*, *qSWT-13-1_ZY_*, *qSWT-13-2_ZY_*, and *qSWT-18-1_ZY_*, were detected in one individual environment with *R*^2^ > 10% (Table 2).

Overall, 38 QTLs were associated with three different seed shape traits in both the K3N and ZY populations; out of them, 20 QTLs have been reported for the first time, while earlier studies have already reported the remaining 18 QTLs (Table 2). Moreover, 17 out of 38 QTLs were major, with *R*^2^ > 10%, and four of them, viz., *qSLW-6-1*_ZY_, *qSLW-20-1*_K3N_, *qSLT-10-1_ZY_*_,_ and *qSLT-20-1*_K3N,_ were detected stably in more than one individual environment. The most prominent major and stable QTL was *qSLW-20-1_K3N_* (novel QTL), with the highest LOD value of 9.01 in an individual environment, identified at 53.61 cM position on Chr20 and expressing a PV of 26.84% (Table 2). The 16 QTLs have positive additive effects with beneficial alleles inherited from KeFeng35, whereas the remaining 22 QTLs possess negative additive effects with favorable alleles derived from Nannong1138-2 (Table 2).

### 2.4. MCIM Mapping and Comparison of CIM and MCIM Methods

To further validate the QTLs detected by CIM, we performed another method of mixed-model-based composite interval mapping (MCIM) to dissect the additive effect QTLs and QTL x E interactions. By using the MCIM method, we identified a total of 48 additive effect QTLs distributed on 15 chromosomes for all six traits related to seed size and shape in both the RIL populations and all three environments, which expressed 1.69 to 29.35% of the PV (Table 3). Moreover, the additive effect of different QTLs was either negative or positive; for example, 30 and 18 QTLs have positive and negative additive effects, respectively. Hence, indicating that both parents contribute beneficial alleles for seed size and shape traits in ZY and K3N populations (Table 3). Out of 48 QTLs, 10 QTLs were significant, with *R*^2^ > 10%, whereas the remaining 38 QTLs were minor, with *R*^2^ < 10% (Table 3).

Among these 48 QTLs, 15 QTLs showed significant additive by environment interaction (AE) effects (Table 3). However, four QTLs viz., *qSL-13-4_ZY_*, *qSW-13-3_ZY_*, *qST-13-4_ZY_*, and *qST-10-1_K3N_* revealed AE effect at all environments, while seven and four QTLs showed AE effect in two and one specific environments, respectively (Table 3). The AE effect of these 15 QTLs associated with seed size and shape traits could express the PV that varies from 0.01 to 4.15%. The remaining 33 QTLs identified through the MCIM approach do not possess any AE effect; hence, they are environmentally stable QTLs (Table 3).

Lastly, we performed a comparative analysis of QTLs detected by CIM and MCIM approaches. A total of 88 and 48 QTLs were identified by CIM and MCIM, respectively. Among these QTLs, 15 QTLs were common and are detected by both methods in the same physical genomic interval, indicating the reliability and stability of these QTLs. Besides, by comparing the physical genomic regions of QTLs identified in both populations (ZY and K3N) and mapping methods (CIM and MCIM), two QTLs, viz., *qSW-1-1_ZY_* and *qSLT-20-1_K3N_*, were detected in common, with *R*^2^ > 10%, identified for the first time. Hence, these QTLs were considered as the most stable and novel QTLs that could be utilized potentially for gene cloning and MAB of soybean seed size and shape traits.

### 2.5. Epistatic Interaction Effects

A total of 16 pairs of epistatic QTLs were detected for seed size and shape in both RIL populations (Table 4). Out of these 16 pairs, four epistatic QTL pairs, viz., *qSL-2-1_K3N_* and *qSL-2-2_K3N_*, *qST-9-1_K3N_* and *qST-12-4_K3N_*, *qSLT-2-3_K3N_* and *qSLT-7-1_K3N_*, and *qSWT-6-1_K3N_* and *qSWT-8-4_K3N_*, possess both significant AA and AAE interaction effects with PV of 1.71–9.70% and 1.68–12.03% expressed, respectively (Table 4). However, the remaining 12 QTLs pairs had only significant AA effects and did not possess any significant AAE interaction effects (Table 4). Hence, the above findings indicate that environment and epistatic interaction effects have considerable influence on the regulation of phenotypic expressions of seed size and shape traits in soybeans. Though, except for three QTLs, viz., *qSL-13-3_ZY_*, *qSL-13-4_ZY_*, and *qSW-13-4_ZY_*, all the remaining additive-effect QTLs did not show any epistatic effects.

### 2.6. Colocalization of QTLs in QTL cluster/Hotspot

**Figure 3:**
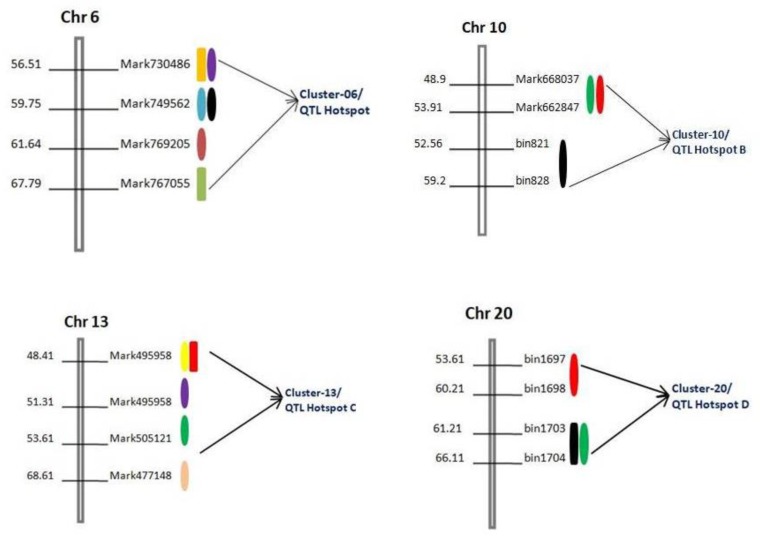
Diagram showing the physical location of four QTL clusters/hotspot regions (cluster-06, cluster-10, cluster-13, and cluster-20) on four different chromosomes viz., Chr6, Chr10, Chr13, and Chr20 identified in two RIL populations across multiple environments Different colors indicate different QTLs within same region.

A QTL cluster/hotspot is defined as a densely populated QTL region of the chromosome that contains multiple QTLs associated with various traits. In this study, we observed colocalization of QTLs on four QTL Clusters/hotspots located on different chromosomes, viz., Chr6, Chr10, Chr13, and Chr20, and were named Cluster-06/QTL Hotspot A, Cluster-10/QTL Hotspot B, Cluster-13/QTL Hotspot C, and Cluster-20/QTL Hotspot D, respectively (Table 5). The highest concentration of QTLs for seed size and shape traits was identified in “QTL Hotspot A” of Chr06, spanning the physical interval of 2.19Mb (Figure 3). This QTL hotspot harbors six QTLs (three major and three minor), viz., *qSW-6-1_ZY_*, *qST-6-1_ZY_*, *qSL-6-1_ZY_*, *qSW-6-2_ZY_*, *qST-6-2_ZY_*, and *qSLT-6-1_ZY_*, associated to seed size and shape traits, expressing a PV of 5.43–15.35% (Table 5). Another set of QTL-rich regions possessing five QTLs (two major and three minor), viz., *qSL-13-1_ZY_*, *qSW-13-1_ZY_*, *qST-13-2_ZY_*, *qST-13-3_ZY_*, and *qSLW-13-1_ZY_* was “QTL Hotspot C”, with a length of 6.3 Mb (Table 5 and Figure 3). However, both “QTL Hotspot B” and “QTL Hotspot D” contain three QTLs each associated with studied traits and spanning the physical interval of 4.0Mb and 2.3Mb expressed PV of 6.60–17.03% and 10.22–26.84%, respectively (Table 5). Furthermore, all these four “QTL cluster/hotspots” comprise many significant QTLs identified in more than one individual environment. QTLs within “QTL Hotspot B” were identified in both ZY and K3N populations (Table 5 and Figure 3). Hence, these four major “QTL hotspots” are the stable genomic regions governing the inheritance of seed shape and size in soybeans.

### 2.7. Candidate Gene Mining within Major “QTL Hotspots”

**Figure 4:**
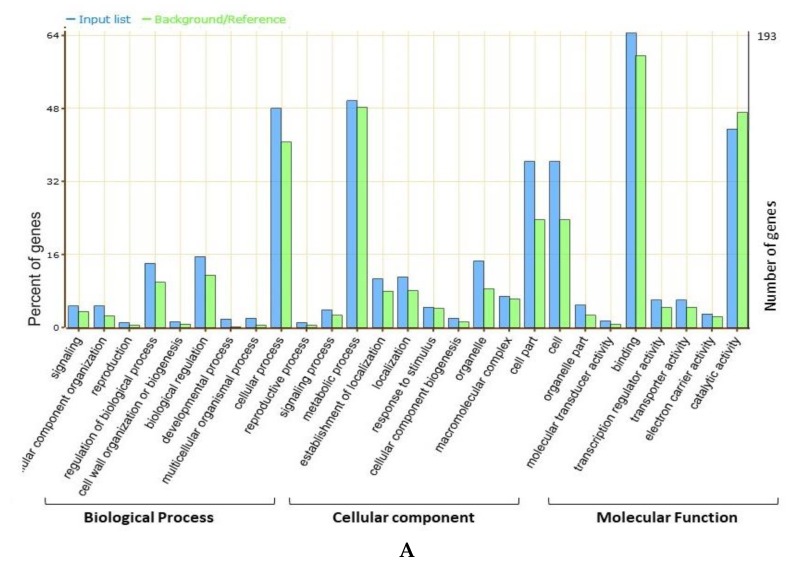

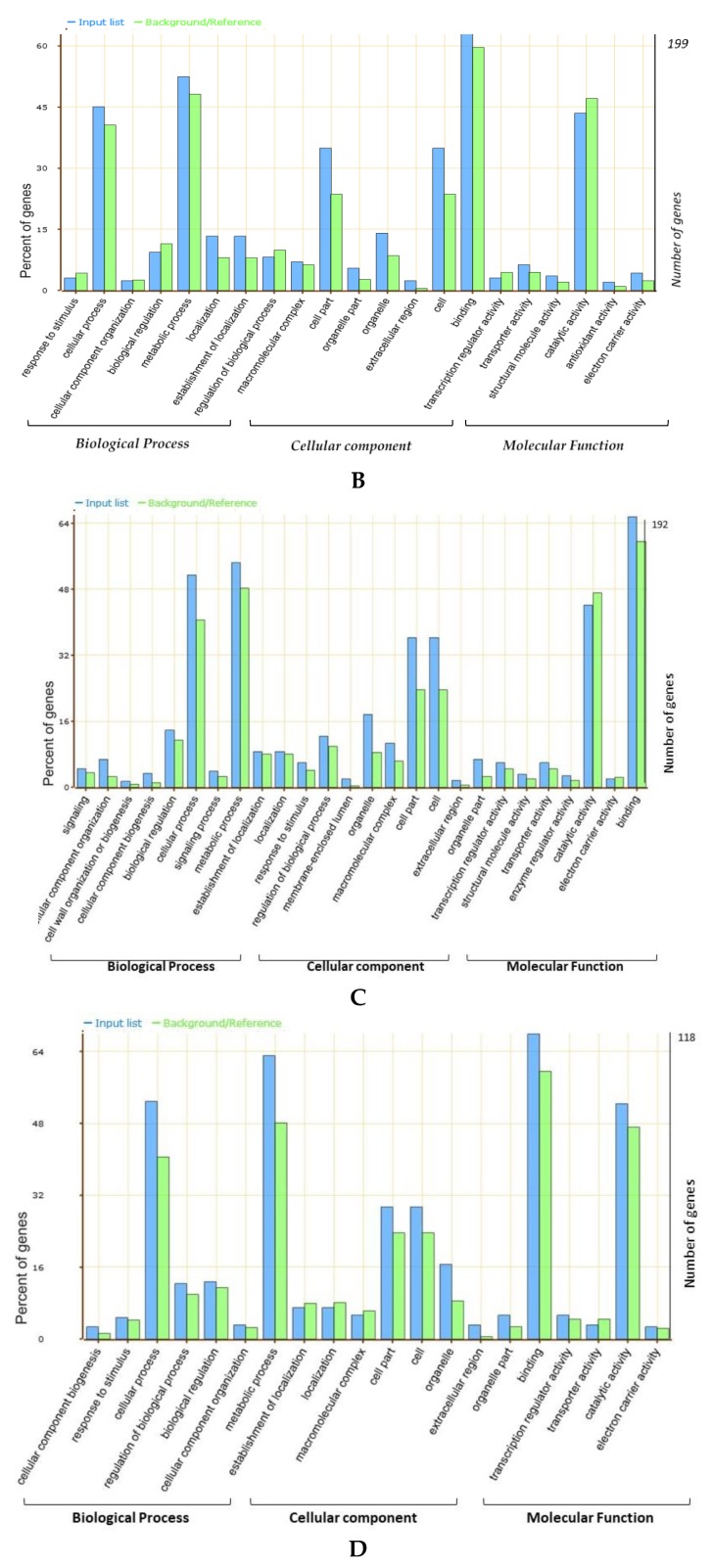
agriGO annotation information. (**A**) Cluster-06 (QTL Hotspot A), (**B**) Cluster-10 (QTL Hotspot B), (**C**) Cluster-13 (QTL Hotspot C), and (**D**) Cluster-20 (QTL Hotspot D).

The whole-genome sequence and gene annotations availability makes it possible to identify possible candidate genes within major genomic regions. In the present study, all the model genes along with their gene annotations were downloaded from Phytozome and Soybase. In total, we identified 2406 gene models within the physical genomic interval of all four major “QTL hotspots” (Table S4). An online web-based toolkit agriGO V2.0 was used for a gene ontology (GO) enrichment analysis to visualize the biological process, molecular function, and cellular component main categories (Figure 4). Among all the genes present within the four “QTL hotspots”, only the 831, 193, 192, and 118 genes from “QTL Hotspot A”, “QTL Hotspot B”, “QTL Hotspot C”, and “QTL Hotspot D”, respectively, had GO annotations available (Figure 4). In all the four major “QTL hotspots”, a higher percentage of genes were associated within the terms cellular process, metabolic process, cell part, cell, catalytic activity, and binding (Figure 4), suggesting a vital role of these terms in regulating seed size and shape in soybeans.

Based on the gene annotations, available literature, and GO enrichment analysis, we predicted 26, 19, 35, and 18 candidate genes from “QTL Hotspot A”, “QTL Hotspot B”, “QTL Hotspot C”, and “QTL Hotspot D,” respectively (Table S6). These genes function directly or indirectly in regulating seed development, as well as seed shape and size, such as mitotic cell division, storage of proteins and lipids, transport, metabolic process, signal transduction of plant hormones, degradation of the ubiquitin-proteasome pathway, and fatty acid beta-oxidation (Table S6). To further refine the above-predicted candidate genes list, we retrieved RNA-Seq data of these candidate genes from Soybase (www.soybase.org) [35].

**Figure 5:**
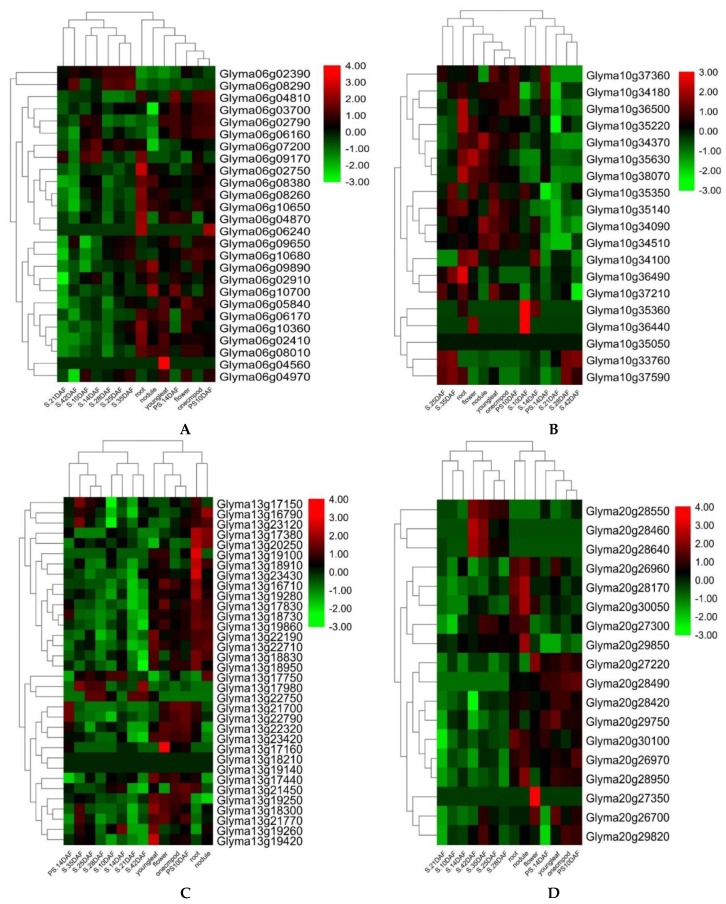
Heat map exhibiting the expression profiles of 23 candidate genes among the different soybean tissues and development stages from four QTL hotspots/clusters. (**A**) Cluster-06 (QTL Hotspot A), (**B**) Cluster-10 (QTL Hotspot B), (**C**) Cluster-13 (QTL Hotspot C), and (**D**) Cluster-20 (QTL Hotspot D). Heat map was generated using the RNA-sequencing data downloaded from online dataset SoyBase. Youngleaf—young leaf, Onecmpod—1 cm of pod, PS—pod shell, DAF—days after flowering, and S—seed.

Based on RNA-seq analysis, 23 genes out of above 88 predicted candidate genes showed significantly higher gene expression/fold-change in the seed development stages, root nodules, leaf, and pod shell. These genes include nine *(Glyma06g02390*, *Glyma06g08290*, *Glyma06g04810*, *Glyma06g03700*, *Glyma06g02790*, *Glyma06g06160*, *Glyma06g07200*, *Glyma06g09650*, *and Glyma06g10700)*; two *(Glyma10g35360 and Glyma10g36440)*; six *(Glyma13g17750*, *Glyma13g17980*, *Glyma13g21770*, *Glyma13g18730*, *Glyma13g21700*, *and Glyma13g22790)*; and six *(Glyma20g28550*, *Glyma20g28460*, *Glyma20g28640*, *Glyma20g27300*, *Glyma20g29750*, *and Glyma20g30100)* genes from “QTL Hotspot A”, “QTL Hotspot B”, “QTL Hotspot C”, and “QTL Hotspot D”, respectively (Figure 5 and Table 6). Hence, these 23 genes might be the possible candidate genes regulating seed size and shape in soybean. However, they need further functional validation to check their actual roles in governing seed size and development.

## 3. Discussion 

Seed shape and size is an economically important trait determining the yield and quality in soybeans. Hence, developing soybean cultivars with improved seed shapes and sizes is considered as a critical objective of soybean breeding programs. However, to develop improved cultivars, it is a prerequisite to have a detailed understanding of genetic architecture, as well as a mechanism underlying the trait of interest. Both seed shape and size are complex quantitative traits, governed by multiple genes and are highly environmentally sensitive. Although, over the past decades, many QTLs related to soybean seed shape/size have been reported but not stable and confirmed, due to small-sized mapping populations and low-density genetic maps, and, hence, not implied for breeding improved seed shapes and sizes in soybean. Thus, the present study aimed to utilize high-density intraspecific linkage maps of ZY and K3N populations, evaluated in three different environments, to identify the stable significant main-effect QTLs, “QTL Hotspots”, and epistatic-effect QTLs, as well as their interactions with the environment; additionally, find possible candidate genes for soybean seed sizes and shape traits. In this study, ANOVA results revealed a significant difference among the RILs of both ZY and K3N populations for all six traits (*P* < 0.01, Table S2). Similar to previous studies, our study also reported that all six traits related to seed size/shape were significantly affected by G, E, and G × E [7,36]. Frequency distribution of all six traits (SL, SW, ST, SLW, SLT, and SWT) showed the characteristics of continuous variations, and all these traits have transgressive segregation in both directions, which indicates that both parents contributed favorable alleles for these traits (Figure 1). These findings are in agreement with the prior findings, which also stated continuous distribution and transgressive segregation for seed size/shape traits among RILs of soybeans in multiple environments [3,17,22]. In our study, the estimated heritability of all six traits was high (>60%) in both RIL populations across all three environments (Table S1), which was consistent with previous studies [7]. The high heritability suggests that if the trial repeated in the same growing/environment conditions, there would be a high possibility of achieving the same kind of phenotypic results. A highly significant correlation (either positive or negative) between any two seed shapes or seed size traits and between seed size and shape traits is in accordance, as previously reported by Xu et al. [5].

QTL mapping is a practical approach and has been frequently employed for the detection of QTLs/genes underlying the quantitative traits in crop plants. However, the efficiency and accuracy of QTL mappings are influenced, mainly by parental diversity and marker density [26]. The quality of genetic maps has a significant impact on the accuracy of QTL detection, and, consequently, increasing marker density can intensify the resolution of QTL mapping [37]. Hence, it is a prerequisite to utilize high-density linkage maps for improving the efficiency and accuracy of linkage mapping and MAS. In this study, high-density genetic maps of ZY and K3N populations were used, consisting of 3255 SLAF and 1733 bin markers, respectively. The markers in both linkage maps, viz., ZY and K3N, were integrated to all 20 LGs and covered the total length of 2144.85 and 2362.44 cM, respectively, with an average distance between adjacent markers of 0.66 cM and 1.36 cM, respectively.

The use of high-density bin-maps assisting in QTL identification with tightly linked markers provided a good foundation for analyzing quantitative traits. However, to reduce environmental errors, RILs were planted in three environments (consisting of different locations and years), and each of the environments was statistically different. Jansen et al. [38] described that the QTL position and effects could be accurately evaluated if the phenotypic data collected in various environments were different from a statistical perspective. Although, markers associated with the QTLs regulating the seed sizes and shapes in soybeans have been mapped on all linkage groups (Soybase, www.soybase.org). However, for cross-validation and improving the accuracy of QTL mapping results, we used two different methods for QTL mapping, viz., CIM and MCIM. A total of 88 and 48 QTLs were detected by CIM and MCIM methods, respectively, associated with all six traits related to seed size and shape (Table 1, Table 2 and Table 3). Among these QTLs, 15 common QTLs were verified through both CIM and MCIM, indicating that these QTLs were stable and utilized effectively as potential candidate regions for enhancing seed sizes and shapes in soybeans. The QTL results of our study revealed better matches with the SoyBase database (www.soybase.org; Table 1 and Table 2); however, 51 (CIM) and 27 (MCIM) QTLs were identified for the very first time (Table 1, Table 2 and Table 3). These novel QTLs collectively expressed more than 90% of PV for seed size and shape, suggesting their potential value for the development of improved soybean cultivars. Among these novel QTLs, *qSL-9-1_ZY_*,*_K3N_*, *qST-6-2_ZY_*, *qSLW-6-1*_ZY_, *qSLW-20-1_K3N_*, *qSLT-10-1_ZY_*, and *qSLT-20-1_K3N_* were reported as stable and major QTLs, identified in more than one individual environment, with *R*^2^ > 10%. Besides, by comparing the physical genomic regions of QTLs identified in both populations (ZY and K3N) and mapping methods (CIM and MCIM), two major and novel QTLs, viz., *qSW-1-3_ZY_* and *qSLT-20-3_K3N_*, were characterized commonly in both mapping methods. These above seven unique and stable QTLs significantly represent potential loci for the improvement of seed sizes and shapes in soybeans. Hence, identification of many new and unique QTLs in the present study suggests distinct genetic architecture in the population derived from the diverse Chinese cultivated soybean genotypes and the need to use more germplasm for revealing the complex genetic basis of soybeans. The favorable alleles for seed size and shape traits were contributed by both parents of two RIL populations, viz., ZY and K3N. Therefore, it is critical to note that not only the higher phenotype parent contributes beneficial alleles but also the contribution of favorable alleles by lower phenotype parents cannot be disregarded; similar results are also described earlier [30].

The stability of the QTL is essential for use in a breeding program. In addition to novel stable QTLs identified for both seed size and shape traits, this study also identified 37 and 21 QTLs through the CIM and MCIM methods, which have been previously colocalized in the same physical interval by earlier studies (see references in Table 1, Table 2 and Table 3). Out of these colocalized QTLs, 12 and 3 QTLs detected by the CIM and MCIM methods were major (*R*^2^ > 10%). Therefore, our results showed the reliability of QTL mapping. Furthermore, these QTLs can be utilized as principal targets to identify the candidate genes and MAS in future studies.

It has been demonstrated that epistatic and QTL by environment interaction effects are the two crucial genetic factors that make an enormous contribution to the phenotypic variation observed in complex traits, and the knowledge of those interaction effects is vital for understanding the genetic mechanism of complex traits [39,40]. Previous studies revealed that the seed sizes and shapes of soybeans is significantly affected by the environment [36]. Moreover, knowledge of specific QTL by environment interactions can guide the search of varieties adapted to particular environments. The QTLs with more significant additive effects are often considered more stable [41,42]. For example, the *qSW-1-1_ZY_* and *qST-18-2_ZY_* (additive effect: 0.14) identified in both CIM and MCIM methods; though, *qSLT-6-5_ZY_* (additive effect: 0.001) was detected only in the MCIM method (ZY only) (Table 3). The genetic architecture of seed size and shape also includes epistatic interactions between QTLs [11,43]. Hence, ignoring intergenic interactions will lead to the overestimation of individual QTL eff*e*cts, and the underestimation of genetic variance [44], consequently, could result in a substantial drop in the genetic response to MAS, particularly at late generations [45]. In the present study, 16 pairs of digenetic epistatic QTLs pairs were identified for seed size and shape in both populations and expressed phenotypic variations that varied from 1.71 to 9.70% (Table 4). Except for *qSL-13-3_ZY_*, *qSL-13-4_ZY_*, and *qST-13-4_ZY_*, all the remaining epistatic QTLs do not possess additive effects alone, suggesting that these loci might serve as modifying genes that interact with other genes to affect the phenotypes of seed sizes and shapes (Table 4). All 16 pairs have significant AA, but only four QTL pairs, viz., *qSL-2-1_K3N_* and *qSL-2-2_K3N_*, *qST-9-1_K3N_* and *qST-12-4_K3N_*, *qSLT-2-3_K3N_* and *qSLT-7-1_K3N_*_,_ and *qSWT-6-1_K3N_* and *qSWT-8-4_K3N_*_,_ hold significant AAE interaction effects. However, the total AAE phenotypic variations expressed by these four epistatic pairs was 19.14%. These results show that epistatic and environmental interactions are fundamental for understanding the genetic basis of seed sizes and shapes in soybeans, demonstrating that these eff*e*cts should be considered in a QTL mapping program and could increase the accuracy of phenotypic value predictions in MAS.

Colocalization of QTLs on chromosomes for different traits related to seed size and shape were also observed in this study. This colocalization of QTLs linked to related traits on chromosomes was reported earlier in soybeans and referred to as “QTL cluster/hotspots” [46]. In this study, we scrutinized a few genomic regions containing QTL clusters and found four QTL clusters/hotspots on four different chromosomes, viz., Chr06, Chr10, Chr13, and Chr20 (Figure 3 and Table 5). The QTLs within each cluster/hotspot are associated with three or more traits related to seed sizes and shapes in soybeans. The highest number of six and five QTLs were observed in “QTL Hotspot A” and “QTL Hotspot C”, respectively, harboring QTLs for more than three traits related to seed size and shape (Figure 3 and Table 5). The other two hotspots, viz., “QTL Hotspot B” and “QTL Hotspot D”, contain three QTLs, each for three different traits related to seed size and shape (Table 5). These QTLs clusters/hotspots have not reported and added to the growing knowledge of the genetic control of these traits. The phenomenon of the QTL clustering might represent a linkage of genes/QTLs or result from the pleiotropic effects of a single QTL in the same genomic region [47]. This colocalization of QTLs for different seed size and shape traits was following the fact that they were highly significantly correlated with each other (Table 1). These “QTL hotspot” regions showed that the QTLs linkage/pleiotropy could facilitate the enhancement of seed size and shape. Previously, some of the QTLs for other traits have also been identified in the same region of “QTL Hotspot A” on chromosome 06, which are related to seed oil and protein content [48,49] and days to flowering [50]. In the case of “QTL Hotspot B”, QTLs related to seed weight and seed yield [51], length of the reproductive stage [33], days to flowering, and maturity [33] were reported in the same physical interval.

Similarly, earlier studies have also reported QTLs for seed weight [7] and seed volume [33] in the “QTL Hotspot C” region on Chr13. In “QTL Hotspot D”, QTLs related to seed maturity [33] and seed oil content [52] have formerly reported. Seed oil and protein content in soybeans have reported a significant correlation with seed size and shape [53], as seed oil and protein content represents a major component of soybean seeds, representing 38–42% and 18–22%, respectively; hence, these traits are directly related to seed sizes and shapes in soybeans [13]. Both seed size and shape are important yield component traits [54] and it has been reported that days to flowering and maturity is directly correlated to yield in soybeans [55,56], signifying the potential probability of common genic factors for these traits and also showing the necessity to promote further study for these regions. These QTL clusters have provided some valuable information to define genome regions associated with different traits. Based on the comprehensive analysis of clusters in this study, breeding programs targeting an increase of seed sizes and shapes with high yield and superior quality can focus on hotspot clustering and select QTLs around the region. Finally, the existence of QTL clusters/hotspots has provided proof that genes related to some crop traits are more densely concentrated in certain genomic regions of crop genomes than others [33,51].

Identification of candidate genes underlying the QTL region is of great interest for practical plant breeding. Earlier studies based on QTL mapping of seed size and shape did not practice mining for candidate genes [22,54], and, to date, only a few seed size/shape-related genes have been isolated from the soybean. For example, the *Ln* gene has a large effect on the number of seeds per pod and seed size/shape [57], and, recently, the *PP2C-1* (protein phosphatase type-2 C) allele from wild soybean accession ZYD7 were found to contribute toward the increase in seed size/shape [58]. Based on the gene annotations, available literature, and GO enrichment analysis, the present study identified the possible candidate genes regulating the seed sizes and shapes in soybeans that underlies the four categorized “QTL hotspots”. Gene ontology (GO) analysis revealed that most of the genes underlying the above four “QTL hotspots” belong to the terms cellular process, metabolic process, cell part, cell, catalytic activity, and binding, and these elements are reported as being vital in seed development [59,60,61]. A total of 2406 gene models were mined within the physical interval of the four “QTL hotspots.” Out of them, 88 were considered as possible candidate genes, based on the GO enrichment analysis, gene function, and available literature. These 88 predicted candidate genes have functions that are directly or indirectly involved in seed development, influencing the shape and size of seeds, such as lipid storage, transport and metabolic processes, signal transduction of plant hormones, degradation of the ubiquitin-proteasome pathway, fatty acid beta-oxidation, the brassinosteroid-mediated signaling pathway, and the auxin biosynthetic process (Table S5). From the available gene expression data (RNA-seq), 23 of the 88 predicted candidate genes expressed significantly higher gene expression, particularly in seed development stages, root nodules, leaf, and pod shell (Figure 5 and Table S5). Out of these 23 genes, five genes, viz., *Glyma06g04810*, *Glyma06g03700*, *Glyma13g17980*, *Glyma13g21770*, and *Glyma20g30100* have functions that are related to seed development, ovule development, endosperm, and embryo development, which have been reported to directly contribute to seed sizes and shapes in crop plants, including soybeans [62,63]. Likewise, *Glyma06g02390*, *Glyma06g06160*, *Glyma06g07200*, and *Glyma13g18730* encode RING/U-box superfamily proteins/protein ubiquitination. The ubiquitin pathway has recently been known to play an essential part in seed size determination [60]. Several factors involved in ubiquitin-related activities have been revealed to determine seed sizes in *Arabidopsis* and rice [60]. Genes, viz., *Glyma06g08290*, *Glyma20g28460*, *Glyma20g28640*, *Glyma20g29750*, *Glyma20g28550*, *Glyma13g22790*, *Glyma20g29750*, and *Glyma20g27300*, function in lipid storage, seed maturation, and cell growth, which have formerly been reported to determine seed size and shape in oilseeds, including soybeans [64]. For example, overexpression of *GmMYB73* promotes lipid accumulation in soybean seeds, which leads to increased seed sizes in soybeans [65]. Genes, viz., *Glyma06g09650*, *Glyma10g35360*, *Glyma10g36440*, *Glyma13g17750*, and *Glyma13g21700*, are involved in auxin biosynthesis, responses to auxin stimulus, and responses to ethylene stimulus. The auxin regulates seed weights and sizes in *Arabidopsis* [22,66]. *Glyma06g10700* functions to regulate the brassinosteroid stimulus, which positively governs seed size [62]. Hence, based on the gene function, GO, and RNA-Seq analysis, the above 23 genes were considered as the most potentially possible candidate genes for regulating the seed sizes and shapes in soybeans. However, it requires further validation and verification to confirm their actual roles in seed sizes/shapes in soybeans, as well as their future uses for the improvement of seed quality traits. Some of these genes were already included in our ongoing project for functional validation to ascertain their effects on the seed sizes and shapes. Hence, the precise identification of QTLs in a specific physical interval through the use of a high-density map would make it easy to identify candidate genes.

## 4. Materials and Methods

### 4.1. Plant Material and Experimental Conditions

In the present study, two related RIL populations, viz., ZY and K3N, consisting of 236 and 91 lines, respectively, were used for elucidating the genetic basis of seed shapes and sizes in soybeans. The ZY and K3N populations were derived through a single seed descent (SSD) method by crossing a common higher seed size parent Nannong1138-2 (N) with two cultivated soybean varieties, viz., Zhengxiaodou (Z) and KeFeng35 (K3), having smaller seed sizes [67]. All the plant material was received from Soybean Germplasm Gene Bank, located at the National Centre for Soybean Improvement (Ministry of Agriculture), Nanjing Agricultural University, Nanjing, China. The F_6:9_–F_6:11_ generations of both RIL populations were planted in three different environments, viz., Jiangpu Experimental Station, Nanjing, Jiangsu Province (Latitude 33°03′ N; Longitude 63°118′ E) in 2012 and 2017 (2012JP and 2017JP) and Fengyang Experimental Station, Chuzhou, Anhui Province (Latitude 32°87′ N; Longitude 117°56′ E) in 2012 (2012FY). Both RIL populations, along with their parents, were planted in a single-line plot of 1 m in length and 0.5 m in width in a randomized complete block design with three replications. In each environment, standard cultural and agronomic practices were trailed, as previously described [68,69].

### 4.2. Phenotypic Evaluation and Statistical Analysis

For the phenotypic assessment of seed size and shape, we collected seeds from the randomly selected ten plants harvested from the middle of each block across three different environments (2012JP, 2012FY, and 2017JP) in both RIL populations. The seed size traits include seed length (SL), seed width (SW), and seed thickness (ST), whereas seed shape was assessed using three different ratios, viz., seed length/seed width (SLW), seed length/seed thickness (SLT), and seed width/seed thickness (SWT). The SL, SW, and ST were measured in millimeters (mm) using the vernier caliper instrument, according to Kaushik et al. [39]. However, SLW, SLT, and SWT were estimated from the individual values of the SL, SW, and ST, respectively, by following Omokhafe and Alika [41].

Descriptive statistics, such as mean, range (maximum and minimum values), coefficient of variation (CV%), skewness, and kurtosis for above seed size and shape traits in both RIL populations, including their parents, were calculated using the SPSS17.0 software (http://www.spss.com) [42]. For each environment, an analysis of variance (ANOVA) was carried out using a generalized linear model (GLM) program of SAS PROC (SAS Institute Inc. v. 9.02, 2010, Cary, NC, USA). The ANOVA for the combined environment (CE) was also performed in SAS software using mixed PROC with random factors: lines, environments, replication within environments, and the line-by-environment interaction. Pearson correlation coefficient (*r*) among traits was calculated from the average data using PROC CORR in combined environments. The broad-sense heritability (*h*^2^) in RIL populations was estimated using the following equation:

(1) $$h^{2}=\sigma_{G}^{2}/(\sigma_{G}^{2}+\sigma_{GE}^{2}/n+\sigma_{e}^{2}/\mathrm{nr})$$

where $\sigma_{G}^{2}$ is the genotypic variance, $\sigma_{GE}^{2}$ is the variance of the genotype-by-environment interaction, $\sigma_{e}^{2}$ is the error variance, *n* is the number of environments, and r is the number of replications within an environment [44].

### 4.3. SNP Genotyping and Bin Map Construction

Genetic map construction began with the extraction of DNA from the young and fresh leaves of both RIL populations, along with their parents, by following the protocol of Zhang et al. [45]. DNA library construction, high-throughput sequencing (RAD-Seq), high-quality SNP acquisition, and SLAF/bin marker integration for ZY and K3N populations, respectively, were performed as described by Huang et al. [70] and Cao et al. [30]. These SLAF and bin markers were employed to develop the linkage maps of the ZY and K3N populations, respectively, using JoinMap 4.0 [71]. High-density genetic maps of the ZY and K3N populations contained 3255 SLAF and 1733 bin markers, respectively. The total length of the ZY and K3N maps were 2144.85 and 2362.44 cM, with an average distance between the adjacent markers as 0.659 and 1.36 cM, respectively (Table S7). The average length of each linkage group was 162.75 and 86.65 cM for ZY and K3N linkage maps, with the mean marker density of each linkage group as 107.24 and 118.122, respectively (Table S7).

### 4.4. QTL Mapping for Seed Size and Shape

For QTL analysis, we used the WinQTLCart 2.5 software [47] and QTLNetwork 2.2 [72]. The model of composite interval mapping (CIM) was used to identify the main-effect QTLs (M-QTLs) with a 10 cM window at a walking speed of 1cM for the WinQTLCart 2.5 software. The LOD threshold was premeditated using 1000 permutations for an experimental-wise error rate of *P* = 0.05 to determine whether the QTL was significantly associated with the traits [73]. The model of mixed linear composite interval mapping (MCIM) was applied to identify significant additive effect QTLs, epistatic QTLs (AA), genotype-by-environment interaction effects (additive by the environment (AE) and AA by the environment (AAE)) in the QTLNetwork 2.2 [74]. The physical location of M-QTLs on each chromosome were drawn by using MapChart 2.1 software [75].

QTLs were named by following standard nomenclature [76], with minor modifications. For example, for the QTL denoted as *qSW-1-1_ZY_*, *q* indicates QTL, *SW* stands for the trait (seed width), -1 show the chromosome on which the QTL detected, -1 also indicates the order of QTL identified on the chromosome for each trait, and_ZY_ represents the ZY-RIL population in which QTL was detected.

### 4.5. Mining of Candidate Genes for Major QTLs

QTLs identified in two or more than two environments with *R*^2^ > 10% were considered as significant and stable QTLs [77]. By utilizing the online resource databases of Phytozome (http://phytozome.jgi.doe.gov) and SoyBase (http://www.soybase.org), we downloaded all the genomic data within the physical interval position of the major “QTL hotspots”, and candidate genes were predicted based on the gene annotations (http://www.soybase.org and https://phytozome.jgi.doe.gov), as well as previously published literature. Gene ontology (GO) information was derived from SoyBase through online resources: GeneMania (http://genemania.org/); Gramene (http://archive.gramene.org/db/ontology); the Kyoto Encyclopedia of Genes and Genomes website (KEGG, www.kegg.jp); and the National Centre for Biotechnology Information (NCBI: https://www.ncbi.nlm.nih.gov). These were used to screen the predicted candidate genes further. Gene ontology (GO) enrichment analysis was conducted for all the genes within the four major “QTL hotspots”, viz., “QTL Hotspot A”, “QTL Hotspot B”, “QTL Hotspot C”, and “QTL Hotspot D”, using agriGO V2.0 (http://systemsbiology.cau.edu.cn/agri-GOv2/) [78]. The freely available RNA-Seq dataset at the SoyBase website was obtained to analyze the expression of predicted candidate genes in different soybean tissues, as well as the development stages. A heat map for the visualization of fold-change in the expression patterns of these predicted candidate genes was constructed by using TBtools_JRE1.6 software [79].

## 5. Conclusions

In conclusion, the present study is a detailed investigation for elucidating the genetic architecture of seed sizes and shapes in soybean. In aggregate, 88 and 48 QTLs were detected through CIM and MCIM, respectively, including 15 common QTLs, with two major (*R*^2^ > 10%) and novel QTLs, viz., *qSW-1-1_ZY_* and *qSLT-20-1_K3N_*. Besides, 51 and 27 QTLs, identified through CIM and MCIM, respectively, were reported for the first time. All identified QTLs were clustered into four major “QTL cluster/hotspots” and represent the major and stable genomic regions governing the inheritance of soybean seed sizes and shapes. Hence, these “QTL hotspot” regions could be of significant consideration for future soybean breeding. Our study predicted 23 genes as the possible candidates, regulating seed sizes and shapes within the genomic region of four “QTL hotspots”; however, they need further functional validation to clarify their actual roles in seed development. Moreover, our results showed that 15 QTLs exhibited significant AE effects, and 16 pairs of QTLs possessed an epistatic effect. However, except for three QTLs, viz., *qSL-13-3_ZY_*, *qSL-13-4_ZY_*_,_ and *qSW-13-4_ZY_*, all the remaining epistatic QTLs showed no main effects. Hence, the hotspot regions and novel significant stable QTLs identified in the present study will be the main focus of soybean breeders for fine mapping, gene cloning, and the MAB of soybean varieties with improved seed quality and yield.

## Figures and Tables

**Table 1:** Main-effect quantitative trait loci (M-QTLs) identified for three seed-size traits (seed length (SL), seed width (SW), and seed thickness (ST)) in ZY and K3N recombinant inbred line (RIL) populations across multiple environments.

Trait	QTL ^a^	Chr (LG) ^b^	Pos (cM) ^c^	LOD ^d^	Add ^e^	*R*^2^(%) ^f^	Confidence Interval (cM) ^g^	Physical Range(bp) ^h^	Env ^i^	Ref ^j^
**SL**	*qSL-4-1_ZY_*	*4 (C1)*	61.81	4.92	0.1	7.21	60.4–82.2	42,941,550–44,864,597	2012FY	[3]
			72.01	3.47	0.09	4.49			2012JP	
			79.41	4.52	0.1	6.3			2017JP	
	*qSL-6-1_ZY_*	*6 (C2)*	23.61	4.12	0.1	5.43	18.7–25.9	5,404,972–7,692,663	2012JP	[3]
	*qSL-6-2_ZY_*	*6 (C2)*	59.51	3.78	0.08	5.18	58.9–66.6	17,259,711–38,704,696	2012FY	[32]
	*qSL-6-3_ZY_*	*6 (C2)*	66.21	6.22	0.12	8.35	65.7–66.7	38,704,696–41,044,201	2012JP	[32]
	*qSL-9-1_ZY,K3N_*	*9(K)*	27.21	4.95	0.1	7.03	24.6–32.3	5,252,918–5,818,109	2017JP	THIS STUDY
			31.11	4.02	0.15	13	30–36.4		2012FY	THIS STUDY
	*qSL-9-2_ZY_*	*9 (K)*	82.81	3.51	−0.08	4.82	79.2–86	38,148,965–40,891,870	2012FY	THIS STUDY
	*qSL-11-1_K3N_*	*11 (B1)*	83.81	4.16	0.14	12.57	77.5–85.5	10,660,406–15,527,096	2012JP	THIS STUDY
	*qSL-13-1_ZY_*	*13 (F)*	48.81	3.5	0.09	5.07	47–49.8	20,463,309–22,44,2989	2017JP	[32]
			48.81	8.14	0.14	11.46	48–49.8		2012JP	
	*qSL-13-2_ZY_*	*13 (F)*	124.41	6.24	0.11	8.89	123.4–124.8	42,740,832–43,643,315	2012FY	[3]
	*qSL-14-1_ZY_*	*14 (B2)*	184.31	4.94	−0.11	7.2	181.8–185.3	19,020,008–26,651,167	2017JP	THIS STUDY
	*qSL-15-1_ZY_*	*15 (E)*	26.31	3.44	0.09	5.05	18.8–37.5	4,951,107–9,734,486	2017JP	THIS STUDY
	*qSL-17-1_K3N_*	*17 (D2)*	101.41	3.8	0.14	11.95	99.2–103.3	38,148,257–39,028,119	2012FY	THIS STUDY
	*qSL-18-1_K3N_*	*18 (G)*	84.31	3.69	−0.13	11.84	83.3–88.8	15,974,989–35,229,774	2017JP	THIS STUDY
	*qSL-20-1_K3N_*	*20 (I)*	61.21	7.19	-0.18	22.64	55.9–67.7	36,184,890–38,300,982	2012JP	THIS STUDY
**SW**	*qSW-1-1_ZY_*	*1 (D1a)*	95.31	6.35	0.09	8.63	89.9–99.5	49,641,073–51,122,075	2012JP	THIS STUDY
	*qSW-2-1_K3N_*	*2 (D1b)*	97.11	5.24	−0.09	17.32	96–102	42,094,237–43,533,158	2017JP	THIS STUDY
	*qSW-4-1_ZY_*	*4 (C1)*	61.81	4.04	0.06	5.85	60.2–65.1	42,941,550–47,127,389	2012FY	[3]
	*qSW-5-1_K3N_*	*5(A1)*	56.31	3.64	0.08	11.26	53.4–61	34,233,479–36,140,865	2017JP	THIS STUDY
	*qSW-6-1_ZY_*	*6 (C2)*	16.31	10.53	0.12	15.35	15.6–16.6	5,651,662–5,975,443	2012JP	[3,32]
	*qSW-6-2_ZY_*	*6 (C2)*	23.31	10.76	0.11	14.45	20.9–24.7	6,147,315–7,6,92,663	2012JP	[32]
	*qSW-8-1_K3N_*	*8 (A2)*	25.61	4.17	−0.1	12.61	20.5–27.8	6,386,731–8,823,572	2012JP	THIS STUDY
	*qSW-9-1_K3N_*	*9 (k)*	29.31	5.22	0.15	17.24	29.2–37	32,901,15–58,181,09	2012FY	THIS STUDY
	*qSW-9-2_ZY_*	*9 (k)*	46.61	3.77	0.07	5.39	44.6–52	21,069,019–30,126,684	2012JP	THIS STUDY
	*qSW-10-1_K3N_*	*10 (O)*	55.21	3.83	−0.1	12.85	52–59.2	32,040,762–38,080,781	2012FY	THIS STUDY
	*qSW-13-1_ZY_*	*13 (F)*	48.41	7.07	0.09	9.59	48–49.1	20,443,593–22,442,989	2012JP	[33]
			51.31	3.57	0.07	5.32	49.8–52.6		2017JP	
	*qSW-13-2_ZY_*	*13 (F)*	124.31	3.57	0.06	5.19	123.5–124.8	42,740,832–43,643,315	2012FY	THIS STUDY
	*qSW-17-1_ZY_*	*17 (D2)*	2.01	6.22	0.08	10.92	0–3.3	33,39,67–2,389,816	2012FY	THIS STUDY
	*qSW-17-2_ZY_*	*17 (D2)*	9.81	5.76	0.08	10.33	5.1–12.1	20,877,60–34,333,86	2012FY	THIS STUDY
**ST**	*qST-1-1_ZY_*	*1 (D1a)*	86.61	4.61	0.09	6.83	82.4–89.6	48,271,814–49,736,597	2012JP	THIS STUDY
	*qST-1-2_ZY_*	*1 (D1a)*	92.81	4.58	0.09	6.17	89.6–98.3	49,7363,57–50,776,854	2012JP	THIS STUDY
	*qST-2-1_K3N_*	*2 (D1b)*	97.11	5.29	−0.11	15.59	93.6–97.8	41,894,158–42,544,803	2017JP	[33]
	*qST-3-1_K3N_*	*3 (N)*	21.31	3.93	0.1	10.98	14.6–23.4	24,562,76–59,471,80	2017JP	THIS STUDY
	*qST-4-1_ZY_*	*4 (C1)*	62.81	3.45	0.07	4.46	58.2–65.1	42,894,734–47,127,389	2012JP	THIS STUDY
	*qST-5-1_K3N_*	*5(A1)*	93.41	4.92	0.13	15.8	91.1–94	38,801,307–39,045,621	2012JP	THIS STUDY
	*qST-6-1_ZY_*	*6 (C2)*	16.31	6.16	0.11	9.68	15.1–16.6	5,651,662–5,975,443	2012JP	[3]
	*qST-6-2_ZY_*	*6 (C2)*	23.61	8.14	0.12	11.53	21.9–26	6,164,792–7,843,,389	2012JP	[3]
			23.61	4.46	0.08	11.12	21.5–26.2		2012FY	
	*qST-6-3 _K3N_*	*6 (C2)*	129.81	4.17	−0.11	12.49	128.3–132.3	49,654,656–50,477,277	2017JP	[3]
	*qST-8-1_K3N_*	*8 (A2)*	13.41	3.44	−0.1	10	7.5–15.5	3,060,492–5,128,185	2012JP	[3]
	*qST-11-1_ZY_*	*11 (B1)*	23.81	5.44	0.09	8.04	23–31.2	10,235,376–15,990,255	2017JP	THIS STUDY
	*qST-12-1 _K3N_*	*12 (H)*	84.01	5.28	0.14	16.67	80.4–86	34,404,607–35,936,212	2012FY	THIS STUDY
	*qST-12-2_K3N_*	*12 (H)*	89.51	6.26	0.14	9	88.5–92.3	35,660,845–36,343,427	2012FY	THIS STUDY
	*qST-12-3_K3N_*	*12 (H)*	96.51	4.32	0.12	13.69	93.1–110.1	36,343,428–38,545,317	2012FY	THIS STUDY
	*qST-13-1_ZY_*	*13 (F)*	19.21	3.61	0.08	6.39	10.1–33	7,974,412–1,484,336	2012FY	THIS STUDY
	*qST-13-2_ZY_*	*13 (F)*	48.41	4.23	0.09	10.28	46.7–9.7	71,87,17–22,442,989	2012JP	[3]
	*qST-13-3_ZY_*	*13 (F)*	51.31	5.17	0.09	7.87	50.1–52.6	22,197,750–23,410,888	2017JP	[33]
			53.61	3.57	0.08	4.86	52.9–55.6		2012JP	
	*qST-16-1_K3N_*	*16 (J)*	77.01	4.8	0.12	14.24	66–78.2	31,905,448–35,735,751	2012JP	[33]
	*qST-17-1_ZY_*	*17 (D2)*	3.01	5.29	0.09	7.63	0.7–3.3	27,02,76–2,389,816	2012FY	THIS STUDY
	*qST-17-2_ZY_*	*17 (D2)*	8.81	5.24	0.1	8.41	3.3–17.4	2,389,537–5,085,098	2012FY	[33]
	*qST-18-1 _K3N_*	*18 (G)*	72.11	5.39	0.13	14.43	73–82	55,571,932–57,042,462	2012FY	THIS STUDY
	*qST-18-2_ZY_*	*18 (G)*	78.01	4.78	0.09	6.74	73.5–82	11,268,490–46,240,347	2012FY	THIS STUDY

a: QTLs detected in different environments at the same, adjacent, or overlapping marker intervals were considered the same QTL; b: chromosome; c: position of the QTL; d: the log of odds (LOD) value at the peak likelihood of the QTL; e: indicates additive; f: phenotypic variance (%) expressed by the QTL; g: 1-LOD support confidence intervals (confidence interval length); h: physical position of QTL; i: environment; and j: references from www.soybase.org.

**Table 2:** M-QTLs identified for three seed-shape traits (seed length-to-width (SLW), seed length-to-thickness (SLT), and seed width-to-thickness (SWT)) in ZY and K3N RIL populations across multiple environments.

Trait	QTL ^a^	Chr (LG) ^b^	Pos (cM) ^c^	LOD ^d^	Add ^e^	*R*^2^(%) ^f^	Confidence Interval (cM) ^g^	Physical Range (bp) ^h^	Env ^i^	Ref ^j^
**SLW**	*qSLW-5-1_ZY_*	*5(A1)*	60.01	3.55	−0.01	4.77	56.6–62.9	39,366,066–41,2966,26	2012FY	THIS STUDY
	*qSLW-5-2_K3N_*	*5(A1)*	92.81	6.73	−0.02	9.15	89.9–93.4	38,337,588–39,465,963	2017JP	THIS STUDY
	*qSLW-6-1_ZY_*	*6 (C2)*	63.61	6.59	0.02	18.68	50.1–65.6	13,274,690–38,704,696	2012FY	[3,32]
			63.61	8.03	0.02	12.17	62.6–65.9		2017JP	
			66.21	5.94	0.02	17.24	57.6–66.7		2012JP	
	*qSLW-6-2_ZY_*	*6 (C2)*	77.01	5.66	0.01	8.15	76.4–77.8	46,087,483–46,232,257	2017JP	[3]
	*qSLW-7-1_K3N_*	*7 (M)*	10.01	4.8	0.02	12.78	7.5–20.1	1,361,954–3,819,224	2017JP	THIS STUDY
	*qSLW-9-1_K3N_*	*9 (K)*	98.41	3.97	0.02	12.97	88.2–102.2	38,138,667–41,052,048	2012FY	THIS STUDY
	*qSLW-10-1_ZY_*	*10 (O)*	53.91	4.64	−0.01	6.6	49–61.7	41,983,494–45,988,221	2017JP	THIS STUDY
	*qSLW-13-1_ZY_*	*13 (F)*	68.61	4.04	0.01	6.44	62.2–70.8	23,963,991–26,852,039	2012JP	[33]
	*qSLW-16-1_K3N_*	*16 (J)*	68.21	6.39	−0.02	16.92	66.5–73.9	31,905,448–33,541,661	2012JP	[33]
	*qSLW-19-1_K3N,ZY_*	*19(L)*	0.01	3.89	0.02	12.25	0–10.8	1–1,939,363	2012FY	THIS STUDY
			5.81	3.55	−0.02	6.09	0–9.6		2017JP	
	*qSLW-20-1_K3N_*	*20 (I)*	53.61	9.01	−0.03	26.84	52.5–55.1	35,924,513–38,138,435	2012JP	THIS STUDY
			60.21	4.64	−0.02	11.64	53.6–64.8		2017JP	
**SLT**	*qSLT-2-1_ZY_*	*2 (D1b)*	63.91	4.04	−0.03	6.45	61.8–65.7	14,715,990–15,293,225	2012FY	[33]
	*qSLT-6-1_ZY_*	*6 (C2)*	29.01	3.7	−0.02	5.27	25.6–30.3	6,779,201–8,789,201	2012JP	[3]
	*qSLT-6-2_ZY_*	*6 (C2)*	62.31	3.92	0.02	5.71	62–65.6	18,806,329–29,376,980	2012JP	[3]
	*qSLT-6-3_ZY_*	*6 (C2)*	70.31	4.71	0.02	6.79	68.6–70.6	39,478,712–42,3014,72	2012JP	[3]
	*qSLT-6-4_ZY_*	*6 (C2)*	82.61	4.98	0.03	7.82	79.3–83.7	47,288,454–48,097,950	2012JP	[3]
	*qSLT-8-1_ZY_*	*8 (A2)*	3.51	5.3	0.03	8.21	0.5–10.3	1,281,677–4,722,531	2017JP	THIS STUDY
	*qSLT-8-2_ZY_*	*8 (A2)*	16.11	3.83	0.02	5.56	13.2–20	4,722,281–8,343,142	2012FY	[3]
	*qSLT-9-1_K3N_*	*9 (K)*	24.31	4.8	−0.03	11.44	22.6–28.6	2,378,279–3,574,689	2012JP	THIS STUDY
	*qSLT-9-2_K3N_*	*9 (K)*	84.31	4.05	0.03	12.62	81.9–88.4	36,947,988–40,302,752	2012FY	THIS STUDY
	*qSLT-10-1_ZY_*	*10 (O)*	53.91	4.55	−0.03	17	48.9–59.4	41,983,494–45,988,221	2012FY	THIS STUDY
			53.91	4.61	−0.02	16.8	49.5–57.8		2012JP	
			53.91	4.96	−0.03	7.4	47.5–60.1		2017JP	
	*qSLT-11-1 _K3N_*	*11 (B1)*	82.61	3.77	0.03	11.7	77.6–84.5	10,660,406–15,086,914	2012FY	THIS STUDY
	*qSLT-13-1_ZY_*	*13 (F)*	16.21	5.44	−0.03	10.29	6.5–23.3	8,857,191–5,270,536	2012FY	[33]
	*qSLT-14-1_ZY_*	*14 (B2)*	57.21	5.29	−0.03	7.31	55.7–69	76,63,93–45,068,56	2012JP	THIS STUDY
	*qSLT-16-1_K3N_*	*16 (J)*	69.61	3.25	−0.05	3.44	68.7–84.1	32,060,131–37,397,385	2012JP	[33]
	*qSLT-17-1_ZY_*	*17 (D2)*	5.11	4.58	−0.03	6.61	4.4–16.4	2,498,772–5,085,098	2012FY	[33]
	*qSLT-20-1_K3N_*	*20 (I)*	64.21	3.82	−0.02	10.22	59–65.5	35,673,231–38,972,972	2017JP	
			66.11	7.28	−0.04	18.54	59.3–71.9		2012JP	THIS STUDY
**SWT**	*qSWT-2-1_K3N_*_, *ZY*_	*2 (D1b)*	63.91	3.89	−0.02	6.4	56.4–65.7	14,715,990–23,379,924	2012FY	[33]
			67.81	3.92	−0.01	5.9	67–70.3		2017JP	
			67.81	5.74	−0.02	8.55	65.7–70.3		2012JP	
	*qSWT-8-1_ZY_*	*8 (A2)*	2.11	3.46	0.01	5.2	0–13.4	1,315,065–8,343,142	2017JP	THIS SYUDY
			2.51	4.73	0.01	10.88	1.2–9.3		2012JP	
	*qSWT-8-2_ZY_*	*8 (A2)*	16.61	3.48	0.02	5.32	13.2–20	4,722,281–8,343,142	2012FY	THIS STUDY
	*qSWT-9-1 _K3N_*	*9 (K)*	71.51	5.44	−0.01	16.29	65.8–72.2	32,505,690–36,079,751	2017JP	THIS STUDY
	*qSWT-10-1_K3N_*	*10 (O)*	87.71	4.11	0.01	12.47	80.7–99.2	40,440,079–44,537,290	2017JP	THIS STUDY
	*qSWT-11-1_K3N_*	*11 (B1)*	103.61	3.78	−0.01	10.89	101.5–105.3	18,952,782–33,288,718	2017JP	THIS STUDY
	*qSWT-12-1_K3N_*	*12 (H)*	87.31	5	−0.02	4.55	85.8–92.4	34,926,974–36,343,427	2012FY	THIS STUDY
	*qSWT-13-1_ZY_*	*13 (F)*	16.21	4.93	−0.02	9.84	6.4–23.4	8,857,191–5,270,536	2012FY	[33]
	*qSWT-13-2_ZY_*	*13 (F)*	29.81	4.26	−0.02	8.07	17.8–37.2	6,777,564–1,267,746	2017JP	[33]
	*qSWT-16-1_K3N_*	*16 (J)*	74.81	4.66	−0.02	13.59	72.7–78.6	33,458,104–35,735,751	2012JP	[33]
	*qSWT-18-1_ZY_*	*18 (G)*	78.71	3.74	−0.02	5.86	72.4–83.7	56,974,254–46,749,768	2012FY	THIS STUDY

a: QTLs detected in different environments at the same, adjacent, or overlapping marker intervals were considered the same QTL; b: chromosome; c: position of the QTL; d: the log of odds (LOD) value at the peak likelihood of the QTL; e: indicates additive; f: phenotypic variance (%) expressed by the QTL; g: 1-LOD support confidence intervals (confidence interval length); h: physical position of QTL; i: environment; and j: references from www.soybase.org.

**Table 3:** Additive and additive x environment interaction effects of QTLs associated with seed shape traits in two RIL populations.

RIL	Trait	QTL	Chr	Pos (cM)	Physical Range (bp)	Flanking Marker	Additive Effect		AE Effect				Ref
							A	PVE (%)	AE1	AE2	AE3	PVE (%)	
**ZY**	**SL**	*qSL-4-2_ZY_*	4	39.22	19283057–19438579	Mark386837–Mark359625	0.18	22.47	NS	NS	NS	0	[3]
		*qSL-6-1_ZY_*	6	65.93	18831802–19024597	Mark750099–Mark741078	0.09	6.04	NS	NS	NS	1.69	[32]
		*qSL-9-2_ZY_*	9	82.03	39583668–39417035	Mark596882–Mark570229	0.08	3.89	NS	NS	NS	0	THIS STUDY
		*qSL-13-3_ZY_*	13	78.75	31540635–31390215	Mark473668–Mark478524	0.08	4.65	−0.03 **	0.03 **	NS	0.82	[3]
		*qSL-13-4_ZY_*	13	4.001	13278146–14133381	Mark471284–Mark486987	0.06	2.62	0.02 **	−0.01 *	−0.01 *	0.45	[3]
		*qSL-14-2_ZY_*	14	12.14	47107927–47737451	Mark941949–Mark890565	−0.08	3.97	0.01 **	NS	−0.01 *	0.11	THIS STUDY
		*qSL-15-1_ZY_*	15	30.54	7595034–8201285	Mark817834–Mark818425	0.07	2.89	NS	NS	NS	0.04	[3]
		*qSL-17-2_ZY_*	17	54.6	13439080–13701008	Mark96769–Mark84717	0.06	2.61	NSN	NS	NS	0	[34]
	**SW**	*qSW-1-2_ZY_*	1	67.22	17489394–20846299	Mark974988–Mark977669	0.03	1.69	NS	0.03 **	−0.04 **	2.78	THIS STUDY
		*qSW-1-1_ZY_*	1	100.2	51095466–51296512	Mark1014325–Mark988734	0.14	29.35	NS	NS	NS	0	THIS STUDY
		*qSW-4-1_ZY_*	4	64.09	48139232–48129734	Mark370122–Mark383650	0.08	9.23	NS	NS	NS	0.1	[3]
		*qSW-4-3_ZY_*	4	13.9	3681724–5115633	Mark411964–Mark375360	0.13	25.78	NS	NS	−0.01 *	0.25	[3]
		*qSW-6-1_ZY_*	6	23.63	6820998–6873235	Mark743934–Mark764418	0.07	6.39	NS	0.05 **	−0.05 **	4.15	[32]
		*qSW-9-3_ZY_*	9	58.19	30576094–33868843	Mark584128–Mark577210	0.04	2.86	NS	NS	NS	0	THIS STUDY
		*qSW-13-3_ZY_*	13	80.04	32094249–32215414	Mark487690–Mark477270	0.09	12.55	−0.01 *	0.03 **	−0.02 **	1.67	THIS STUDY
		*qSW-17-3_ZY_*	17	26.12	5914087–5923952	Mark80080–Mark102449	0.07	6.28	0.03 **	NS	−0.02 **	1.4	[3]
		*qSW-20-1_ZY_*	20	82.14	41039353–41758780	Mark244793–Mark230802	0.04	2.48	NS	NS	−0.01 *	0.16	THIS STUDY
	**ST**	*qST-1-3_ZY_*	1	66.15	24564849–25434623	Mark988529–Mark966630	0.06	3.58	NS	NS	NS	0	THIS STUDY
		*qST-4-2_ZY_*	4	44.13	30867521–32458924	Mark404804–Mark410274	0.06	3.43	0.01 **	NS	−0.02 **	0.47	[3]
		*qST-6-4_ZY_*	6	28.98	8734791–8864415	Mark778253–Mark768262	0.09	7.87	NS	NS	NS	0	THIS STUDY
		*qST-13-4_ZY_*	13	74.5	29909876–29933390	Mark492165–Mark480651	0.08	6.47	−0.04 **	0.02 **	0.01 **	1.31	[33]
		*qST-17-3_ZY_*	17	26.12	5914087–5923952	Mark80080–Mark102449	0.08	5.27	NS	NS	NS	0.03	[3]
		*qST-18-2_ZY_*	18	73.25	11452216–56974484	Mark107824–Mark1041506	0.14	17.36	NS	NS	NS	0	THIS STUDY
		*qST-20-1_ZY_*	20	46.1	32964872–34278811	Mark222598–Mark260922	0.09	6.68	NS	NS	NS	0	[33]
	**SLW**	*qSLW-6-3_ZY_*	6	68.61	39116570–39478973	Mark735467–Mark752696	0.02	16.59	NS	NS	NS	0.02	[33]
		*qSLW-9-2_ZY_*	9	26.46	5253206–5593340	Mark586124–Mark605515	0.02	6.23	NS	NS	NS	0.01	THIS STUDY
		*qSLW-10-1_ZY_*	10	34.22	40118012–41534124	Mark647482–Mark674118	−0.01	3.59	NS	NS	NS	0.22	THIS STUDY
		*qSLW-14-1_ZY_*	14	2.62	46512262–46896487	Mark910347–Mark906347	−0.03	3.59	NS	NS	−0.01	0.22	THIS STUDY
	**SLT**	*qSLT-2-2_ZY_*	2	54.96	13892893–14031565	Mark45679–Mark24395	−0.04	14.3	NS	NS	NS	0	THIS STUDY
		*qSLT-6-5_ZY_*	6	72.26	43230405–43114475	Mark747633–Mark770074	0	7.18	NS	NS	NS	0	[34]
		*qSLT-8-3_ZY_*	8	107.06	44543723–45326890	Mark457232–Mark423205	0.03	8.95	NS	NS	NS	0	THIS STUDY
		*qSLT-10-1_ZY_*	10	34.22	40118012–41534124	Mark647482–Mark674118	−0.03	8.65	NS	NS	NS	0	THIS STUDY
		*qSLT-10-2_ZY_*	10	3.31	4454862–4625724	Mark637694–Mark659817	0.03	9.56	NS	NS	NS	0	[34] -
		*qSLT-14-2_ZY_*	14	118.74	27709972–27836278	Mark905511–Mark937850	−0.02	4.16	NS	NS	NS	0	THIS STUDY
		*qSLT-17-1_ZY_*	17	12.06	3433165–1091815	Mark91398–Mark70303	−0.02	3.77	NS	NS	NS	0	[33]
		*qSLT-18-1_ZY_*	18	72.71	11452216–56974484	Mark107824–Mark1041506	−0.04	3.77	NS	NS	NS	0	THIS STUDY
	**SWT**	*qSWT-2-2_ZY_*	2	45.8	10992717–11277233	Mark4308–Mark33823	−0.04	3.72	NS	NS	NS	0	THIS STUDY
		*qSWT-8-3_ZY_*	8	111.68	45326630–45114110	Mark466182–Mark457232	0.01	4.91	NS	NS	NS	0	THIS STUDY
		*qSWT-13-3_ZY_*	13	118.22	39676002–42053780	Mark492087–Mark510247	−0.01	4.03	NS	NS	NS	0.36	[34]
		*qSWT-14-1_ZY_*	14	132.78	43567951–44159326	Mark927308–Mark890844	−0.01	3.64	NS	NS	NS	0.01	THIS STUDY
		*qSWT-18-1_ZY_*	18	73.25	56974254–11452436	Mark107824–Mark1041506	−0.03	18.77	NS	NS	NS	0.79	THIS STUDY
**K3N**	**SL**	*qSL-17-3_K3N_*	17	118.01	40207655–41906774	bin1466–bin1467	0.08	5.71	NS	NS	NS	0	THIS STUDY
		*qSL-20-1_K3N_*	20	55.91	36184890–36777026	bin1698–bin1699	−0.09	6.72	NS	−0.01 *	0.03 **	1.02	THIS STUDY
	**ST**	*qST-10-1_K3N_*	10	59.22	36682803–37647030	bin827–	−0.08	7.05	−0.03 **	0.02 **	0.02 **	2.06	THIS STUDY
						bin829							
	**SLW**	*qSLW-5-2_K3N_*	5	94.46	38132148–38801307	bin402–	−0.01	7.06	NS	NS	NS	0.01	[34]
						bin403							
		*qSLW-16-1_K3N_*	16	69.6	32318950–33186025	bin1362–bin1363	−0.01	2.2	NS	NS	NS	0.02	[34]
	**SLT**	*qSLT-16-1_K3N_*	16	75.68	33853674–35244129	bin1371–bin1372	−0.04	19.15	NS	−0.01 *	NS	1.49	THIS STUDY
		*qSLT-20-1K3N*	20	68.27	37878839–38300982	bin1704–bin1705	−0.03	11.77	NS	NS	NS	0.05	THIS STUDY

Chr.: chromosome. * *p* < 0.05 and ** *p* < 0.01. PVE indicates phenotypic variation expressed by additive effects. AE1: 2012FY, AE2: 2012JP, and AE3: 2017JP.

**Table 4:** Estimated epistatic effects (AA) and environmental (AAE) interactions of QTLs for seed shape and size traits across all environments.

RIL	QTL_i	Chr_i	Pos_i	Marker Interval_i	QTL_j	Chr_j	Pos_j	Marker Interval_j	(AA) Effect		(AAE) Effect			
									AA	PVE (%)	AE1	AE2	AE3	PVE (%)
**ZY**	*qSL-13-3_ZY_*	13	78.75	Mark473668–Mark478524	*qSL-13-4_ZY_*	13	4.001	Mark471284–Mark486987	−0.05	1.84	NS	NS	NS	-
	*qST-1-4_ZY_*	1	67.22	Mark962092–Mark962281	*qST-13-5_ZY_*	13	118.5	Mark492087–Mark510247	−0.07	4.16	NS	NS	NS	-
	*qST-1-5_ZY_*	1	88.72	Mark1006029–Mark981292	*qST-13-4_ZY_*	13	32.59	Mark489890–Mark487004	−0.07	4.11	NS	NS	NS	0.02
	*qST-6-5_ZY_*	6	26.19	Mark738100–Mark750615	*qST- 9-1_ZY_*	9	98.04	Mark591384–Mark570193	−0.07	4.96	NS	NS	NS	0.06
	*qSLT-6-6_ZY_*	6	11.85	Mark729845–Mark775156	*qSLT-10-3_ZY_*	10	14.29	Mark628845–Mark623926	0.02	3.98	NS	NS	NS	-
	*qSLT-15-1_ZY_*	15	26.29	Mark799922–Mark817834	*qSLT-19-1_ZY_*	19	64.16	Mark114395–Mark141336	0.03	9.59	NS	NS	NS	-
	*qSWT-1-1_ZY_*	1	67.22	Mark990284–Mark986367	*qSWT-13-4_ZY_*	13	83.91	Mark484073–Mark489301	0.02	6.7	NS	NS	NS	0.25
	*qSWT-13-5_ZY_*	13	53.11	Mark505121–Mark508857	*qSWT-20-1_ZY_*	20	83.56	Mark250253–Mark257473	0.02	6.2	NS	NS	NS	0.01
**K3N**	*qSL-2-1_K3N_*	2	40.91	bin87–bin88	*qSL-2-2_K3N_*	2	95.94	bin130–bin131	−0.11	9.7	−0.05 **	0.01 **	NS	3.02
	*qST-9-1_K3N_*	9	71.51	bin748–bin749	*qST-12-4_K3N_*	12	0.6	bin962–bin963	0.04	1.71	NS	0.03 **	NS	2.41
	*qSLW-4-1_K3N_*	4	50.29	bin285–bin286	*qSLW-15-1_K3N_*	15	148.55	bin1298–bin1299	0.03	4.34	NS	NS	NS	-
	*qSLW-7-2_K3N_*	7	37.3	bin526–bin527	*qSLW- 12-1_K3N_*	12	55.09	bin990–bin991	−0.01	6.33	NS	NS	NS	0.08
	*qSLT-2-3_K3N_*	2	4.2	bin164–bin165	*qSLT-7-1_K3N_*	7	94.74	bin568–bin569	−0.01	3.21	NS	0.01 **	NS	1.68
	*qSWT-6-1_K3N_*	6	97.8	bin470–bin471	*qSWT-8-4_K3N_*	8	109.71	bin672–bin673	−0.04	7.56	−0.01 *	NS	0.01**	12.03
	*qSWT-11-2_K3N_*	11	97.12	bin934–bin935	*qSWT-17-1_K3N_*	17	5.47	bin1386–bin1387	0.01	7.1	NS	NS	NS	1.11
	*qSWT-11-3_K3N_*	11	106.76	bin952–bin953	*qSWT-14-2_K3N_*	14	45.84	bin1170–bin1171	0.01	6.48	NS	NS	NS	0.74

Chr_i and Chr_j indicate the two sites involved in epistatic interactions and Pos indicates genetic position for each of the sites. * *p* < 0.05 and ** *p* < 0.01. PVE indicates phenotypic variation expressed by epistatic effects. AE1: 2012FY, AE2: 2012JP, and AE3: 2017JP.

**Table 5:** Four QTL hotspots/clusters detected in ZY and K3N RIL populations across multiple environments.

QTL Cluster Name	Chr_Bin Range	QTL Name	Physical Range (bp)	LOD	Additive Effect	R^2^ (%)
**Cluster-06/QTL Hotspot A**	Chr06_Mark730486-Mark767055(ZY)	*qSW-6-1_ZY_*	5651662–7843389	10.53	0.12	15.35
		*qST-6-1_ZY_*		6.16	0.11	9.68
		*qSL-6-1_ZY_*		4.12	0.1	5.43
		*qSW-6-2_ZY_*		10.76	0.11	14.45
		*qST-6-2_ZY_*		8.14	0.12	11.53
				4.46	0.08	11.12
		*qSLT-6-1_ZY_*		3.70	−0.02	5.27
**Cluster-10/QTL Hotspot B**	Chr10_ Mark668037-Mark662847(ZY) Chr10_bin821-bin828(K3N)	*qSLT-10-1_ZY_*	41983494–45988221	4.55	−0.03	17.03
				4.61	−0.02	16.83
				4.96	−0.03	7.40
		*qSLW-10-1_ZY_*		4.64	−0.01	6.60
		*qSW-10-1_K3N_*		3.83	−0.1	12.85
**Cluster-13/QTL Hotspot C**	Chr13_ Mark477148-Mark495958(ZY)	*qSL-13-1_ZY_*	20463309–26852039	3.5	0.09	5.07
				8.14	0.14	11.46
		*qSW-13-1_ZY_*		7.07	0.09	9.59
				3.57	0.07	5.32
		*qST-13-2_ZY_*		4.23	0.09	10.28
		*qST-13-3_ZY_*		5.17	0.09	7.87
				3.57	0.08	4.86
		*qSLW-13-3_ZY_*		4.04	0.01	6.44
**Cluster-20/QTL Hotspot D**	Chr20_bin1697-bin1704(K3N)	*qSLW-20-1_K3N_*	35957343–38300982	9.01	−0.03	26.84
				4.64	−0.02	11.64
		*qSL-20-1_K3N_*		7.19	−0.18	22.64
		*qSLT-20-1_K3N_*		3.82	−0.02	10.22
				7.28	−0.04	18.54

**Table 6:** Predictive gene annotation information.

Cluster/QTL Hotspot	Mapped IDs	Gene Functional Annotation
**Cluster-06/QTL Hotspot A**	*Glyma06g02390*	RING/U-box superfamily protein/protein ubiquitination
	*Glyma06g08290*	Lipid storage
	*Glyma06g04810*	Seed coat development extracellular region
	*Glyma06g03700*	Seed development ovule development
	*Glyma06g02790*	Response to auxin stimulus
	*Glyma06g06160*	Ubiquitin-protein ligase activity
	*Glyma06g07200*	Response to ethylene stimulus ubiquitin-protein ligase activity
	*Glyma06g09650*	microtubule nucleation, response to auxin stimulus, cytokinin mediated signaling pathway
	*Glyma06g10700*	Response to brassinosteroid stimulus sterol biosynthetic process
**Cluster-10/QTL Hotspot B**	*Glyma10g35360*	Response to auxin stimulus cellular component
	*Glyma10g36440*	Auxin biosynthesis
**Cluster-13/QTL Hotspot C**	*Glyma13g17750*	Response to auxin stimulus protein dimerization activity
	*Glyma13g17980*	Embryo development
	*Glyma13g21770*	Endosperm development embryo development
	*Glyma13g18730*	Ubiquitin-dependent protein catabolic process
	*Glyma13g21700*	Response to ethylene stimulus-response to auxin stimulus
	*Glyma13g22790*	Protein kinase activity
**Cluster-20/QTL Hotspot D**	*Glyma20g28550*	Seed maturation protein
	*Glyma20g28460*	Lipid storage
	*Glyma20g28640*	Lipid storage
	*Glyma20g27300*	Lipid metabolic process seed maturation cell growth
	*Glyma20g29750*	Multidimensional cell growth polysaccharide biosynthetic process regulation of hormone levels
	*Glyma20g30100*	Embryo development seed development protein phosphorylation

## Supplementary Materials

The following are available online at https://www.mdpi.com/1422-0067/21/3/1040/s1, Table S1: Descriptive statistics, broad-sense heritability (h2) of Seed Shape & size traits evaluated in two recombinant inbred lines (RILs) ZY & K3N. The RILs and the parent were grown in different field environments of years and locations. * &** represent significance at 5% and 1%, respectively; Table S2: Combined analysis of variance (ANOVA) for Seed shape & size trait (SL,SW,ST,SLW,SLT,SWT) in ZY & K3N RIL Populations across three different environments (2012FY, 2012JP and 2017JP); Table S3: Correlation analysis among six Seed size/shape traits (SL,SW,ST,SLW,SLT,SWT). * &** represent significance at 5% and 1%, respectively; Table S4: Model genes within cluster-06, cluster-10, cluster-13 and cluster-20 regions in both RIL Populations (ZY & K3N); Table S5: Candidate genes within cluster-06, cluster-10, cluster-13 and cluster-20 in ZY and K3N-RIL Populations and their relative expression data retrived from RNA-seq data available in soybase; Table S6: Predicted candidate genes within cluster-06, cluster-10, cluster-13 and cluster-20 regions in both RI Populations (ZY & K3N) based on known functional annotation; Table S7: Distribution of SLAF and bin markers mapped on soybean chromosomes/linkage groups in ZY & K3N Populations, respectively.

## Abbreviations

SL	Seed Length
SW	Seed Width
ST	Seed Thickness
SLW	Seed Length-to-Width
SLT	Seed Length-to-Thickness
SWT	Seed Width-to-Thickness
G x E	Genotype x Environment
QTL	Quantitative Trait Loci
USDA	United States Department of Agriculture
SSRs	Simple Sequence Repeats
M-QTL	Main-Effect QTL
E-QTL	Epistatic-Effect QTL
RIL	Recombinant Inbred Line
PV	Phenotypic Variation
SSD	Single Seed Decent
RCBD	Randomized Complete Block Design
Mm	millimeters
CV	Coefficient of Variation
ANOVA	Analysis of Variance
GLM	Generalized Linear Model
CE	Combined Environment
MSG	Multiplexed Shotgun Genotyping
SNP	Single Nucleotide Polymorphisms
CIM	Composite Interval Mapping
LOD	Logarithm of the Odds
